# Effect of Integrated Internet-Based Acceptance and Commitment Therapy and Behavioral Activation Among Ethnic Minority Young Adults With Alcohol Use Disorder in Hong Kong: Pilot Randomized Controlled Trial

**DOI:** 10.2196/83896

**Published:** 2026-05-21

**Authors:** Getaneh Mulualem Belay, Katherine Ka Wai Lam, Qi Liu, Ting Mao, Yim Wah Mak, Ka Yan Ho

**Affiliations:** 1 School of Nursing, The Hong Kong Polytechnic University, 11 Yuk Choi Road, Hung Hom, Kowloon, Hong Kong, China, +852 27666417; 2 School of Nursing, College of Medicine and Health Sciences, University of Gondar, Gondar, Ethiopia

**Keywords:** acceptance and commitment therapy, alcohol addiction, alcohol use disorder, behavioral activation, ethnic minority young adults

## Abstract

**Background:**

Alcohol use disorder (AUD) profoundly affected ethnic minority young adults due to unique stressors, such as acculturation challenges and discrimination. Our prior studies found that experiential avoidance behavior and discrimination significantly contributed to AUD. These findings warranted third-wave interventions, such as acceptance and commitment therapy (ACT). Nonetheless, barriers to engagement with value-based activities may affect ACT efficacy. Behavioral activation (BA) may be an alternative for this shortcoming. Integrating these interventions has shown promise. However, no studies evaluated its effectiveness in this group. Therefore, evaluating the preliminary effectiveness and feasibility of this intervention was needed.

**Objective:**

This study aimed to evaluate the feasibility measures. The secondary objective aimed to examine its effectiveness on cumulative abstinence duration, drinking days, drinks per drinking day, heavy drinking days, alcohol abstinence self-efficacy, readiness to change, psychological flexibility (Acceptance and Action Questionnaire—version II), and everyday discrimination score.

**Methods:**

A pilot parallel randomized controlled trial was conducted. Forty young ethnic minorities who can speak English and have AUD based on *DSM-5* (*Diagnostic and Statistical Manual of Mental Disorders* [Fifth Edition]) criteria were recruited and randomly assigned to either Acceptance and Commitment Therapy with Behavioral Activation (ACT-BA; n=20) or Treatment-as-Usual (TAU; n=20) using a computer-generated random number; assessors were blinded to group assignment. Outcomes were collected at baseline (T0) and postintervention (T1). The analysis included descriptive statistics, independent samples *t* test, chi-square test, and generalized estimating equations with multiple imputations. To further supplement findings, a qualitative interview was conducted.

**Results:**

A total of 148 ethnic minority young adults were screened, with an eligibility rate of 38.5% (57/148). Of these, 85.9% (49/57) consented, and among them, 81.6% (40/49) were randomized to either the ACT-BA or TAU. The retention rate was 82.5% (33/40), of which 75% (30/40) completed the postintervention assessment (ACT-BA: 16/20, 80%; TAU: 14/20, 70%). The adherence rate was 81.7% (4.9/6 sessions), and participants reported no adverse effects. Finally, 40 participants (20 for each group) were analyzed. The intervention group showed a promising improvement in drinking days (*B*=−4.12, 95% CI −8.10 to −0.13; *P*=.04, *d*=−0.57), drinks per drinking day (*B*=−1.56, 95% CI −3.06 to −0.07; *P*=.04, *d*=−1.89), alcohol abstinence self-efficacy (*B*=11.95, 95% CI 0.10-23.81; *P*=.048, *d*=0.81), and Acceptance and Action Questionnaire—version II (*B*=−6.41, 95% CI −12.77 to −0.06; *P*=.04, *d*=−0.65).

**Conclusions:**

This study, unlike existing evidence, presents an innovative integration of ACT and BA delivered via an internet-based self-help format. The findings contribute to the field by providing preliminary evidence that this integrated intervention is feasible and promising for AUD. The main implication in the real world is to conduct a fully powered randomized controlled trial to further examine its effectiveness with longer follow-up to serve as a stand-alone treatment option for ethnic minorities.

## Introduction

### Background

Alcohol use disorder (AUD) is a severe medical problem characterized by an inability to control alcohol consumption despite adverse physical, psychological, and social consequences [1-6]. Early alcohol consumption accelerates AUD development, leading to academic decline, injuries, and premature mortality among young adults aged 20‐39 years, accounting for 13.5% of global alcohol-related deaths in this age group [7-15].

AUD remains a significant public health issue, disproportionately affecting ethnic minority young adults compared with the general population [16-18]. In Hong Kong, ethnic minorities constitute about 8.4% of the population [19,20]. However, unlike those with the majority ethnicity, that is, Hong Kong and/or Chinese populations, ethnic minorities encounter various challenges, including stigma, discrimination, acculturation stress, low self-esteem, social integration difficulties, and loneliness, contributing to unhealthy drinking behaviors and AUD [7,21-27]. Previous studies showed that over 50% of ethnic minorities have less access to education and employment than the Chinese population, and about 60% of Chinese respondents believed that ethnic minorities should be limited to low-skilled occupations [28]. Our previous empirical evidence further identifies cultural factors, family influences, hedonistic motives, curiosity, low-risk perception, coping motives, social pressures, and subjective cravings as reasons behind AUD development among young adult ethnic minorities [29].

In this regard, existing research has evaluated several psychosocial interventions for AUD [30-36], including motivational interviewing [30,37-39], integrated family and cognitive behavioral therapy [40], combined motivational enhancement therapy and cognitive behavioral therapy [41], common elements treatment approaches [42], guided self-change [35], and home-based ecologically based family therapy [36]. However, our systematic review and meta-analysis revealed limited efficacy of these approaches, highlighting the need for other, more effective treatments [43]. Consistent with this knowledge gap, our qualitative findings demonstrated that ethnic minority adults with AUD frequently engaged in experiential avoidance, using alcohol to cope with acculturation stress, anxiety, depression, and discrimination. This pattern is further corroborated by our cross-sectional study, which identified discrimination experiences and avoidance coping behaviors as significant contributors to AUD. Together, these maladaptive behaviors underscore the challenge in achieving a sustained behavioral change. Consequently, our findings indicate a need for third-wave therapies that promote acceptance of such negative internal experiences [44-47]. Third-wave therapies are a kind of psychosocial intervention focusing on accepting rather than changing negative thoughts and emotions [48].

Acceptance and commitment therapy (ACT) has emerged as a promising intervention for substance use and mental health disorders, enhancing psychological flexibility [45,49]. Grounded in Relational Frame Theory [48,50,51], ACT enhances conscious behavioral adaptation aligned with personal values while accepting negative thoughts and emotions [52]. Unlike traditional therapies, ACT focuses on behavioral change over symptom reduction, making it particularly suitable for avoidance-driven conditions such as AUD [48,51,53]. Literature review suggests that ACT may be a viable treatment option for ethnic minority young adults with AUD [47,54,55].

However, our review identified methodological limitations in existing studies on the effectiveness of ACT on AUD and substantial challenges in engagement and adherence to ACT [56-58]. First, many studies lacked appropriate control groups and failed to directly assess alcohol-related outcomes (eg, drinking days, drinks per drinking day, heavy drinking days, and abstinence duration). Second, for the adherence issues, participants showed low engagement with value-based activities for guiding committed action toward a meaningful life [56,57]. This is potentially attributed to difficulty in identifying enjoyable, rewarding alcohol-free activities, and overemphasis on internal experiences (motions or thoughts) without sufficient attention to overt behaviors. Finally, ACT does not contain any daily activity for practice, which is a critical challenge for ethnic minorities with AUD to identify pleasurable alternatives to replace drinking, leading to relapse despite quitting attempts. Consequently, these limitations warrant integrating novel strategies into ACT to enhance engagement and adherence.

Behavioral activation (BA) may be a possible method to overcome engagement and adherence problems in ACT. BA is a concept from behaviorism [59,60] and is developed from a concept that behavior can directly affect emotions for better or worse [59]. Hence, individuals can put themselves in a situation that is likely to foster positive emotions. For example, a positive event such as listening to uplifting music can lead to happiness and vice versa. BA is a widely recognized and theoretically sound approach that has been applied to treat depression [61,62]. Its application has been extended to substance use disorders (SUDs) via providing rewarding experiences in daily life, different from substance use, thus achieving abstinence and preventing relapse [63-65]. Evidence suggests that BA is a promising intervention for SUD as a stand-alone treatment or being integrated with other therapeutic modalities. The emphasis on rewarding experiences in BA exactly addresses the low engagement and adherence of ACT due to the failure to identify enjoyable, rewarding, and positively reinforcing activities in the intervention process. Because of the distinctive benefits of BA, it appears to be a treatment component complementary to the shortcomings of ACT, thus enhancing the intervention effectiveness if they both are integrated into the treatment protocol. Also, BA is a brief intervention that can be delivered within 4 weeks, which will not significantly prolong the duration of each intervention session, ensuring the participants’ adherence and engagement. In sum, ACT simply encourages an individual to ACT, which includes *A*ccepting the reactions and being present, *C*hoosing a valued direction, and *T*ake actions, while BA motivates an individual to take real actions. Two studies have integrated BA into ACT to manage different conditions in various populations, including an internet-based BA and acceptance-based treatment for depression [66], and an open trial of a new acceptance-based behavioral treatment for major depression and psychotic features [64]. The results were promising, which supported that the integration is not only theoretically sound but also feasible and, importantly, can translate into observable benefits.

Regarding the mode of intervention delivery, our qualitative findings revealed significant treatment barriers among ethnic minority young adults, in which no participant had accessed treatments for AUD due to their engagement in work. Apart from work, their time and financial constraints also hindered them from seeking hospital-based care [26]. Internet-based interventions may be a viable option to address these challenges by enhancing accessibility [67-69], reducing stigma [70], minimizing work disruption [71], and improving cost-effectiveness [68,72,73]. The ACT’s efficacy for depression further supports this modality [72,74]. However, no studies have evaluated internet-based self-help interventions integrating ACT and BA for these populations. Therefore, this study examined the preliminary effectiveness and feasibility of an internet-based self-help–integrated ACT and BA intervention in improving alcohol use abstinence among ethnic minority young adults with AUD.

### Theoretical Framework

The integration of ACT and BA is grounded in principles of functional contextualism [75]. ACT derives from Relational Frame Theory [76,77]. ACT enhances psychological flexibility through the 6 ACT core processes (ie, acceptance, cognitive defusion, being present, self as context, values, and committed action). These processes represent 6 facets of 1 diamond, and the diamond itself is psychological flexibility. Psychological flexibility is defined as the capacity to persist or change one’s behavior to be open and accepting of personal thoughts and feelings, to appreciate and adapt to different situations, and respond to situations that facilitate one’s values and goals [51,76]. Each process contributes to psychological flexibility for AUD treatment as follows: (1) *Being present*: Individuals with AUD often drink alcohol in response to cravings and emotional triggers. By learning the present moment, they can mindfully notice these triggers and create a space for acceptance rather than avoidance. (2) *Acceptance*: This involves opening and making room for such painful feelings, sensations, urges, and emotions that trigger drinking. Instead of struggling with these experiences, individuals learn to allow them to come and go, just like cars driving past outside our house, reducing the urge to escape through drinking. (3) *Cognitive defusion*: Individuals with AUD may be hooked on thoughts like “I need a drink to cope.” Defusion helps them step back and watch thoughts for what they are—nothing more or less than words or pictures. (4) *Self-as-context*: This process assists individuals in recognizing that they are more than their thoughts and feelings and makes individuals aware of whatever they are thinking, feeling, and sensing. (5) *Values*: AUD leads individuals away from what is important to them. Clarifying values helps identify what matters to them in their lives. (6) *Committed action*: This is taking effective actions, guided by our values. For people with AUD, it refers to committing to value-congruent activities despite pain and discomfort.

**Figure 1.**
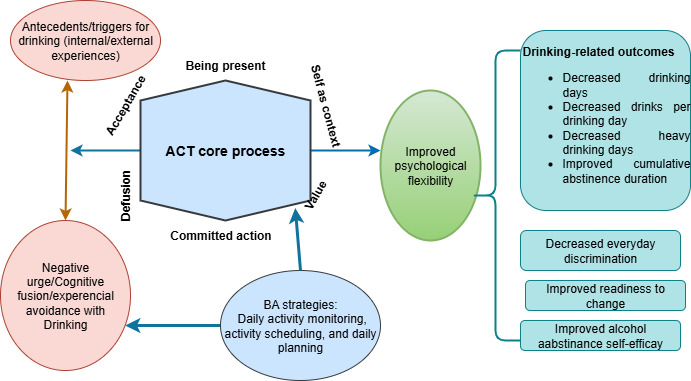
Theoretical framework for integrated acceptance and commitment therapy and behavioral activation interventions. ACT: acceptance and commitment therapy; BA: behavioral activation.

BA is derived from the behavioral theory of substance use [78]. According to this theory, substance use persists partly because of the limited access to competing rewarding activities and a lack of positive reinforcement from behaviors that do not involve substance use. To tackle AUD by BA, individuals have to identify adaptive alternatives to drinking that provide comparable rewards (eg, exercise for stress relief) without adverse consequences. The integration of ACT and BA provides a synergetic efficacy. ACT encourages patients to change their relationship with triggering factors rather than avoiding them, and creates a metacognitive space to tolerate distress, for example, acceptance of cravings [77], while BA complements ACT by increasing engagement in value-congruent activities that compete with drinking [75] and provides a structured method (eg, scheduling exercises and social events) to modify external behavior, that is, alcohol use [79]. For example, a client who values health and connection might replace bar visits with an alcohol-free gathering. This integrated approach targets both internal and external factors that drive AUD [75]. ACT equips individuals with skills to accept negative emotions, whereas BA provides a concrete strategy to disrupt reinforcement cycles and build healthy habits. Together, they provide a sustained psychological flexibility [75] (Figure 1).

### Objectives

This study examined the preliminary effectiveness and feasibility of an internet-based self-help program integrating ACT and BA on alcohol abstinence among Hong Kong ethnic minority young adults with AUD. The primary objective of this study was to evaluate the intervention’s feasibility in terms of various feasibility outcomes under CONSORT (Consolidated Standards of Reporting Trials) 2025, including rates of eligibility, consent, randomization, adherence, retention, completion, missing data, and adverse events. The secondary objective was (1) to evaluate the preliminary effectiveness of this internet-based self-help program integrating ACT and BA on several outcomes, including drinking days, drinks per drinking day, heavy drinking days, cumulative abstinence duration (CAD), alcohol abstinence self-efficacy (AASE), psychological flexibility, readiness to change, and everyday discrimination in this population; and (2) to estimate the effect size of the Acceptance and Commitment Therapy with Behavioral Activation (ACT-BA) intervention on these same outcomes.

## Methods

### Patient and Public Involvement

Due to limited time and the online mode of self-help intervention, patients were not involved in designing and developing the research question, nor in reporting the findings. However, experts participated in developing and reviewing the intervention protocol.

### Trial Design

A single-blinded randomized controlled trial (RCT) was conducted among Hong Kong ethnic minority young adults with AUD from September 15, 2024, to May 15, 2025. This study was used according to the protocol. There were no changes to the trial design, participants, analysis, and outcomes after the study commenced.

### Trial Setting

This study was conducted in Hong Kong, a special Chinese administrative region located east of the Pearl River Delta. In Hong Kong, approximately 7.5 million people live on a land area of 1104 square kilometers [80], of which about 619,552 (8.4%) are ethnic minorities [19].

### Study Participants

#### Eligibility Criteria

Participants who met the following criteria were considered for this study: (1) young adults (aged 18-35 years) from ethnic minorities in Hong Kong, (2) proficient in English (read, write, and speak), (3) voluntary participants, (4) possessing internet-enabled smartphones or electronic devices, and (5) meeting the *DSM-5* (*Diagnostic and Statistical Manual of Mental Disorders* [Fifth Edition]) criteria for AUD, defined as exhibiting at least 2 of the 11 symptoms specified in the DSM-5 [81]. Ethnic minorities in this age range were chosen as it aligns with the US Census Bureau classification for young adults [82], and this age group is the population group with the highest vulnerability for SUDs [83].

#### Exclusion Criteria

Participants were excluded if they met any of the following criteria: (1) they were currently experiencing other psychiatric and severe mental health problems, or actively involved in suicide attempts; (2) planned to leave Hong Kong during the intervention period; (3) had no and/or lack of access to a smartphone and/or other electronic devices; and (4) had prior engagement in ACT and/or BA for AUD.

#### Sample Size

For this pilot study, a sample size of 40 participants (20 per group) was determined through methodological and practical considerations. This is consistent with guidelines suggesting 12‐30 participants per group as adequate for generating meaningful preliminary data [84]. Previous evidence from comparable studies demonstrates feasibility and provides preliminary effect size estimation with similar sample sizes [57,85]. As it was a pilot pre-post study, the final analysis was performed once the full sample size was reached; an interim analysis was not planned.

#### Randomization

Stratified block randomization was implemented by a trained research assistant (RA) to allocate participants into intervention and control groups (20 per group). To ensure baseline balance of prognostic factors, particularly ethnicity, sex, and disease severity, which could confound the intervention effects if unevenly distributed, participants were first stratified by these factors. Within each stratum, block randomization (1:1 ratio, block size of 4) was performed using a computer-based random number generator.

#### Allocation Concealment and Blinding

Allocation sequences were concealed using a computer-generated random number. Due to the nature of the intervention and self-administered questionnaires, blinding of participants was not feasible. However, the outcome assessor was blinded to the participants’ group assignments throughout the study.

#### Recruitment and Data Collection

An RA was responsible for participant recruitment. The RA received a 1-day training regarding study details and data collection procedures from the principal investigator. Participants were recruited in the community through face-to-face approaches at bars, communal recreational areas, schools, and gardens where ethnic minorities usually gather. The RA approached potential ethnic minorities, introduced the study, and gave them a poster to read the details, objectives, and eligibility criteria of the study. Interested ethnic minorities were instructed to scan the QR code and complete a screening in a Google form to determine their eligibility. A brief session was conducted to guide the participants to access the intervention using their smart devices during participant recruitment. Eligible ethnic minorities who agreed to participate gave written consent. The participants’ contact addresses, including email, WhatsApp number, WeChat number, and/or Telegram account, were taken for further connections.

Participants were then provided a personal link to access the Qualtrics website. They were provided instructions to complete baseline data. Consequently, participants were randomly assigned to either the ACT-BA group or the Treatment-as-Usual (TAU) group. Quantitative data were collected at 2 time points, that is, baseline and postintervention. Then, a semistructured interview was conducted among the intervention group after the postintervention assessment.

#### Semistructured Interview

The interviewer contacted all participants in the intervention group for the semistructured interview. However, only 13 participants consented and were invited to the interview immediately after the postintervention assessment. Data saturation was achieved upon interviewing the 13th participant; this was the point at which no additional themes were identified. A male research student conducted interviews face-to-face and/or by phone using a semistructured interview guide on four areas: (1) general experience, (2) intervention content, (3) delivery mode and duration, and (4) perceived usefulness. The interview took an average of 15 minutes. Findings might provide pertinent and supplementary data to enhance the understanding of the intervention feasibility and effectiveness.

#### Intervention

The intervention group received an internet-based self-help program that integrated both ACT and BA, using an online platform in Qualtrics, through a personalized link. The intervention contained a total of 6 weekly individual sessions structured sequentially. An RA in the team sent text reminders to the participants via different information communication systems, for example, WhatsApp and WeChat, 1 day before the intervention session to ensure their attendance. For session 1, it aimed to establish the therapeutic relationship with the participants and assess the participants’ experiential avoidance behaviors. In this session, participants learned through multimedia resources what avoidance behavior was; how it impacted their life in terms of different perspectives, including family, health, work, social life, and the community; and withdrawal symptoms. They also identified alcohol-free rewarding activities and initiated daily monitoring through rating enjoyment or importance (from 1 to 10) in a workbook. High-scoring activities were considered for further value-driven planning. Session 2 focused on value identification, in which participants learned and identified core values across life domains and developed corresponding value-driven activity plans using a structured worksheet. They scheduled at least 1 value-congruent activity per domain. Session 3 introduced the concept of acceptance and cognitive defusion. Participants learned and practiced various acceptance and diffusion skills and mindfulness exercises to manage experiential avoidance, which was usually managed by drinking. They learned different techniques of acceptance by (1) letting feelings, urges, and thoughts occur without a desire to act on them; (2) noticing their strengths while acknowledging their shortcomings; and (3) facing the difficulty without running away from or avoiding it. They also learned defusion skills by considering their thoughts as thoughts only that could come and go, and sometimes their impulsive thoughts about drinking were not facts. Session 4 focused on present moment awareness via mindful practices. Participants learned to observe internal experiences (such as cravings, negative emotions, and thoughts) related to past or future events that led to AUD. Observing self-skills was also developed through metaphors. Particularly, the participants learned how to consider themselves and hence understood that there was a part of them who could think, feel, notice, and observe their cravings and feelings toward drinking. Session 5 reinforced value-directed BA, with participants identifying new value-congruent alcohol-free activities and integrating them into actionable daily plans and taking committed action toward their life goals. Session 6 emphasized the posttreatment plan and relapse prevention strategies. Previous sessions were reviewed, and another long-term goal congruent with their values was developed. However, participants have not received any additional concomitant care or intervention outside of the study protocol from the researcher.

#### Control Group

The control group, referred to as TAU, received standard or usual community care without any active interventions from the study. Particularly, TAU did not contain any standardized, structured psychoeducation, counseling, or concomitant care from the researcher. However, participants continued to receive routine health care, self-help, or any community services that they had already engaged in, and they were asked to report the received services to the research team during the study period. Participants were also provided referrals to specialist care when needed. In previous studies, such standards have been used as a benchmark for comparison with ACT [86-88].

#### Intervention Integrity

The intervention integrity was maintained in several ways. First, the same RA was responsible for updating, reminding, and sending the intervention link to the participants. Second, the RA was trained for 7 days on how to manage online intervention using Qualtrics and follow the intervention process. Third, the intervention content was adapted from the empirically validated ACT manual and BA strategies, with expert validation done by a panel that contained 2 associate professors, experienced in research and delivery of different psychosocial interventions, particularly ACT, an assistant professor with experience in research of addiction and substance abuse, and 3 social workers in an NGO providing services for ethnic minorities in Hong Kong. Fourth, regular supervision and process monitoring were conducted to ensure the smoothness of the implementation. Fifth, all procedures of implementation were systematically documented for transparency.

#### Contamination

Study participants were recruited at various points, and the intervention was provided online through each participant’s account. Participants should not know each other to exchange information. Therefore, the possibility of information sharing seemed unlikely in this study.

### Outcomes

#### Feasibility Outcomes

The feasibility of this study was assessed by evaluating eligibility rate (number of eligible participants divided by number of screened ethnic minorities), consent rate (number of participants consented divided by number of eligible participants), randomization rate (number of participants who were randomized divided by number of participants who consented), adherence rate (number of participants in the intervention group who completed all intervention sessions divided by the number of participants randomized into the intervention group), retention rate (number of participants who remained in the study divided by the number of participants who were randomized), completion rate (number of participants who answered the questionnaires divided by the number of questionnaires being distributed), adverse events that were defined as unfavorable and unintended events that were absent from baseline or appeared to worsen from baseline during the study period, and missing data (the percentage of missing in the dataset).

#### Effectiveness Outcomes

##### Drinking-Related Outcomes

The Timeline Follow-Back Questionnaire was used to assess the following drinking-related outcomes, including drinking days, drinks per drinking day, heavy drinking days, and CAD. Every participant was asked to report the number of standard drinks (1 unit contains 12 grams of pure alcohol) consumed within 30 days pre- and postintervention. Then, the average number of drinks per drinking day, numbers of drinking days and heavy drinking days, and cumulative abstinence were computed. Heavy drinking day is defined as having 4 or more drinks for females, and 5 or more drinks on any day for males [89]. Abstinence refers to the total number of days a participant abstains from alcohol use postintervention [87]. The CAD was calculated by dividing the number of abstinent days by 30 days.

##### Alcohol Abstinence Self-Efficacy

The self-efficacy of participants in alcohol abstinence was measured using the AASE Scale. This scale contains 20 items rated on a 5-point Likert scale, ranging from 0 to 80. Higher scores indicate high self-efficacy to abstain from alcohol use. In this study, the Cronbach α value of this scale was 0.91.

##### Readiness to Change

The participant’s readiness to change in drinking was measured using the Readiness to Change Questionnaire, a 12-item instrument [90]. The internal consistency of the scale was good.

##### Psychological Flexibility

The Acceptance and Action Questionnaire—version II was used to measure psychological flexibility. This questionnaire contains 7 items rated on a 7-point rating scale, from 1 (never true) to 7 (always true) [91]. Higher scores indicate greater levels of psychological inflexibility. This scale showed a good internal consistency (Cronbach α=0.779) in our previous studies conducted among ethnic minority young adults in Hong Kong [92].

##### Everyday Discrimination

The participants’ everyday discrimination was measured using the short version of the Everyday Discrimination Scale [93]. The scale comprises 5 items, rated on a 6-point scale, ranging from 5 to 36 (1=almost never, 6=almost always). They also identified perceived causes of discrimination from options including religion, ethnicity, age, gender, sexuality, disability, or an open-ended category, that is “other.” A higher score on the Everyday Discrimination Scale indicates a frequent experience of discrimination. This scale revealed an excellent internal consistency in our previous cross-sectional study, with a Cronbach α of 0.963 [92].

### Statistical Methods

The data were analyzed using the IBM SPSS (version 23; IBM Corp). Descriptive statistics were used to summarize the social-demographic characteristics of participants and the feasibility measures, including eligibility rate, consent rate, randomization rate, adherence rate, retention rate, completion rate, percentage of missing data, and adverse events. The baseline and posttest outcome variables were checked for normality assumption and the homogeneity of variance using the Shapiro-Wilk and Levene tests, respectively. Mean and SD were used for continuous variables with a normal distribution, while medians and IQRs were used for non–normal distributions. Comparability of participants between groups was examined by independent samples *t* test or Mann-Whitney *U* test for continuous variables, and chi-square test or Fisher exact test for categorical variables. Additionally, paired *t* test (for normally distributed data) and Wilcoxon signed-rank test (non–normally distributed data) were used to examine within-participant mean differences.

Missing data were handled using intention-to-treat (ITT) analysis, considering all participants enrolled and randomized to either 1 of the 2 groups. Little Missing Completely at Random (MCAR) test was used to determine whether the missingness was completely at random or not. Sensitive analyses were also conducted to compare results from per-protocol (PP) (complete-case data) and ITT analyses.

To compare pre-and posttest outcomes, we used the Generalized Estimating Equations (GEE) under the assumption that data missing were completely at random. Additionally, an independent samples *t* test was performed as a supplementary analysis to evaluate a pre-post change in outcomes. However, to preserve statistical power and reduce bias under the assumption that data were missing at random, GEE with multiple imputations (MIs) was used due to the presence of substantial missing data (>5% missingness in posttest measures). The pooled estimates of MIs were used to compute the analysis. If the data were missing at random, a weighted GEE analysis was used, and the effect of time × group assignment interaction on outcome variables was determined.

Parameter estimations for signs of instability were calculated using the quasi-likelihood estimation method integrated within the GEE procedure of IBM SPSS. Quasi-Information Criterion values were used to select the appropriate correlation structure. Model convergence was examined across all imputations and found that they converged successfully without warnings or errors. Intervention effects were estimated based on the difference in pre-post difference using the estimation-based approach for multiple comparisons, that is, we emphasized 95% CI and effect sizes rather than relying solely on *P* values to assess the precision and clinical significance of the intervention effects. The effect size was calculated to assess the clinical significance of the outcomes for between-group comparisons of change scores and was interpreted using Cohen *d*, as follows: small=0.2, moderate=0.5, and large=0.8. Finally, the findings were reported following the CONSORT 2025 guideline [94].

### Qualitative Data Analysis

Regarding the qualitative data, a thematic method of analysis was used. First, all audio recordings were transcribed into English verbatim. A descriptive phenomenological design was chosen to explore the experiences of participants regarding the intervention. Two researchers (GMB and KYH) carefully read transcripts and listened to audio multiple times to understand the participants’ experiences. They independently highlighted the key quotes and labeled them with codes. Any disagreement between coders was resolved through discussion until a consensus was reached. Senior researchers (KYH and YWM) were consulted if a consensus was not reached. Similar codes were grouped into subthemes and eventually themes, and all 13 participants reviewed the findings to ensure that the findings matched with their experiences. Finally, the results were reported following the COREQ (Consolidated Criteria for Reporting Qualitative Research) [95].

### Ethical Considerations

Ethical approval was obtained from the Human Subjects Ethics Subcommittee of the Hong Kong Polytechnic University (reference number HSEARS20240712003). The protocol of this study was submitted on August 28, 2024, and was subsequently published on January 15, 2025, on ClinicalTrials.gov with National Clinical Trial number NCT06779006. This record is available online at ClinicalTrials.gov. Informed written consent was obtained from each participant before the intervention. To ensure confidentiality and privacy, all participants’ data were kept confidential, anonymous, and stored on a secure password-protected server; no personally identifiable information was used in the study. Each participant has been compensated for costs to their time and internet usage; that is, each of them received a 300 Hong Kong dollars (approximately US $38.46) gift card with an exchange rate of 1 US $=7.8 Hong Kong dollars. Finally, there were no supplementary materials or images containing identifiable information for participants in the manuscript. They were informed of their right to decline or withdraw from the study at any time.

## Results

### Participant Characteristics

The study participants’ sociodemographic characteristics are presented in Table 1. Participants had a mean age of 32.43 (SD 2.57) years, with 80% (32/40) female and 87% (35/40) as Filipino; 85% (34/40) were Catholic, 52.5% (21/40) were single, and 85% (34/40) were employed. Of those being employed, 87.5% (35/40) were domestic helpers. Approximately 60% (24/40) attained primary or secondary education, and 32.5% (13/40) cohabited with partners. The median duration of residence in Hong Kong was 5 years, with an IQR of 2‐6. Regarding the AUD severity, 47.5% (19/40) had moderate AUD, 32.5% (13/40) had severe AUD, and 20% (8/40) had mild AUD. Baseline comparisons between groups revealed no significant differences in demographics and the AUD severity.

**Table 1.** Baseline sociodemographic characteristics of ethnic minority young adults with AUD^a^ in Hong Kong (N=40): a pilot randomized controlled trial.

Variables	ACT-BA^b^ (n=20)	TAU^c^ (n=20)	Total (N=40)	*P* value
Age (years) mean (SD)	32.6 (1.98)	32.25 (3.09)	32.43 (2.57)	.68^d^
Sex, n (%)				
Female	16 (40)	16 (40)	32 (80)	≥.99^e^
Male	4 (10)	4 (10)	8 (20)	
Ethnicity, n (%)				
Filipino	17 (42.5)	18 (45)	35 (87.5)	≥.99^e^
Whites	1 (2.5)	1 (2.5)	2 (5)	
African	2 (5)	1 (2.5)	3 (7.5)	
Religion, n (%)				
Catholic	15 (37.5)	19 (47.5)	34 (85)	.25^e^
Orthodox Christians	3 (7.5)	1 (2.5)	4 (10)	
Others	2 (5)	0 (0)	2 (5)	
Marital status, n (%)				
Single	12 (30)	9 (22.5)	21 (52.5)	.40^e^
Married/cohabiting	4 (10)	8 (20)	12 (30)	
Separated/widowed	4 (10)	3 (7.5)	7 (17.5)	
Educational status, n (%)				
≤12 grades (below senior secondary)	11 (27.5)	13 (32.5)	24 (60)	.52^f^
College and above	9 (22.5)	7 (17.5)	16 (40)	
Employment, n (%)				
Unemployed	3 (7.5)	3 (7.5)	6 (15)	≥.99^e^
Employed	17 (42.5)	17 (42.5)	34 (85)	
Occupation, n (%)				
Domestic helper	17 (42.5)	18 (45)	35 (87.5)	≥.99^e^
Daily laborer/others	3 (7.5)	2 (5)	5 (12.5)	
Living arrangements, n (%)				
Living alone	5 (12.5)	1 (2.5)	6 (15)	.25^e^
Living with parents/relatives	4 (10)	5 (12.5)	9 (22.5)	
Living with nonrelatives	7 (17.5)	5 (12.5)	12 (30)	
Living with partners	4 (10)	9 (22.5)	13 (32.5)	
Duration of residence in Hong Kong				
Years, median (IQR)	4.5 (4)	5 (5)	5 (4)	.50^g^
*DSM-5*^h^ criteria for AUD, n (%)				
Mild AUD	5 (12.5)	3 (7.5)	8 (20)	.75^e^
Moderate AUD	8 (20)	11(27.5)	19 (47.5)	
Severe AUD	7 (17.5)	6 (15)	13 (32.5)	

a AUD: alcohol use disorder.

b ACT-BA: Acceptance and Commitment Therapy with Behavioral Activation.

c TAU: Treatment-as-Usual.

d Independent samples *t* test.

e Fisher exact test.

f Chi-square test.

g Mann-Whitney *U* test.

h *DSM-5*: *Diagnostic and Statistical Manual of Mental Disorders* (Fifth Edition).

### Baseline Data

Independent samples *t* test was used to compare the baseline outcomes between the 2 groups (Shapiro-Wilk test, *P*>.05; Levene test, *P*>.05). There was no significant difference between the intervention and control groups regarding the baseline outcome measures (Table 2).

**Table 2.** Baseline outcome measure of ethnic minority young adults with AUD^a^ in Hong Kong: a pilot randomized controlled trial between groups, ACT-BA^b^ (n=20) vs TAU^c^ (n=20).

Baseline outcome	Total (N=40), mean (SD)	ACT-BA (n=20), mean (SD)	TAU (n=20), mean (SD)	Mean difference (95% CI)	*P* value
AASE^d^	47 (14.78)	44.45 (10.79)	49.55 (17.85)	−5.1 (−14.54 to 4.34)	.28
AAQ-II^e^	25.27 (9.83)	24.45 (9.01)	26.1 (10.76)	−1.65 (−8.00 to 4.70)	.60
DDD^f^	5.09 (0.71)	5.16 (0.75)	5.03 (0.68)	.13 (−0.33 to 0.59)	.57
EDS^g^	20.93 (8.50)	19.5 (7.75)	22.35 (9.15)	2.38 (−6.37 to 3.95)	.30
RTCQ^h^	35.88 (9.69)	37.55(8.26)	34.20 (10.90)	3.35 (−2.84 to 9.54)	.46
DD^i^	9.73 (7.20)	9.35 (6.93)	10.10 (7.62)	−0.75 (−5.41 to 3.91)	.67
HDD^j^	6.28 (4.19)	6.55 (5.38)	6.00(2.64)	0.55 (−2.16 to 3.26)	.35
CAD^k^	20.27 (7.20)	20.65 (6.93)	19.90 (7.62)	0.75 (−3.91 to 5.41)	.67

a AUD: alcohol use disorder.

b ACT-BA: Acceptance and Commitment Therapy with Behavioral Activation.

c TAU: Treatment-as-Usual.

d AASE: Alcohol Abstinence Efficacy Scale.

e AAQ-II: Acceptance and Action Questionnaire—version II.

f DDD: drinks per drinking day in the past 30 days.

g EDS: Everyday Discrimination Scale.

h RTCQ: Readiness to Change Questionnaires.

i DD: drinks per day in the past 30 days.

j HDD: heavy drinking days in the past 30 days.

k CAD: cumulative abstinence durations.

### Feasibility of Intervention

**Figure 2.**
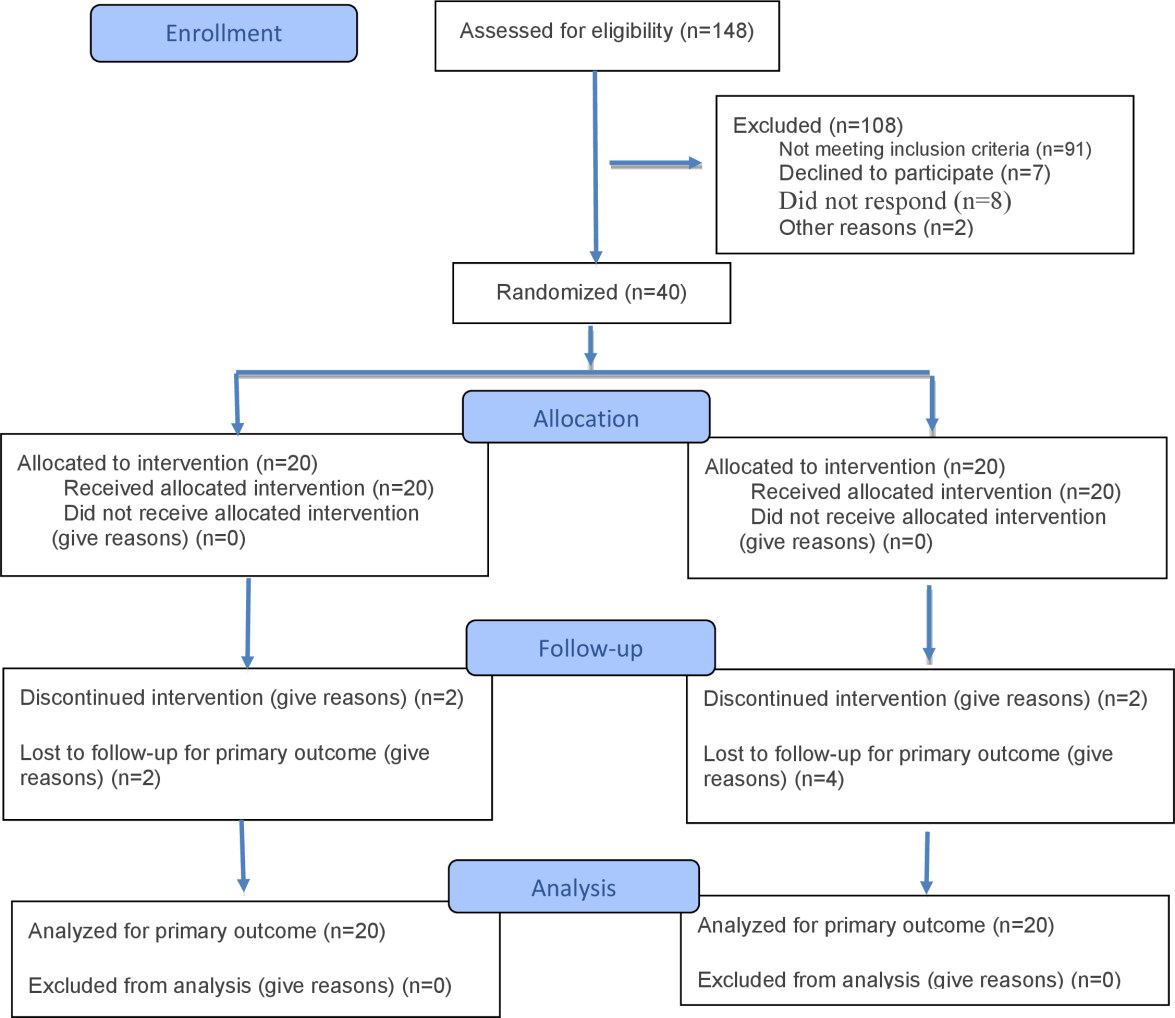
CONSORT (Consolidated Standards of Reporting Trials) 2025 participants flow diagram. The process through the phases of a randomized trial of 2 groups (ie, enrollment, intervention allocation, follow-up, and data analysis).

Figure 2 presents the CONSORT 2025 flow diagram. A total of 148 ethnic minority young adults who drank alcohol were approached from the community. After screening, 38.5% (57/148) met the eligibility criteria. Of them, 85.96% (49/57) consented to join the study. Among these, 81.6% (40/49) were randomly assigned either to the ACT-BA (n=20) or TAU (n=20) group. The retention rate was 82.5% (33/40), with 85% (17/20) in the ACT-BA group and 80% (16/20) in the TAU group. The attrition rate was 15% (3/20) for ACT-BA and 20% (4/20) for TAU, but there was no significant difference in attrition between groups (*P*=.47). Of those who remained in the study, 30 completed the postintervention test, providing a completion rate of 75% (30/40), with 80% (16/20) in the ACT-BA group and 70% (14/20) in the TAU group (Table 3). Participants in the ACT-BA group completed an average of 4.9 out of 6 sessions (adherence rate: 81.7%). No adverse events were reported at postintervention. The trial was not ended or stopped early. The median session duration was 30 (IQR 35) minutes. The median interval between sessions was 7 (IQR 2) days.

**Table 3.** Adherence to intervention among ethnic minority young adults with alcohol use disorder in Hong Kong: a pilot randomized controlled trial.

Sessions	ACT-BA^a^ group (n=20)		TAU^b^ group (n=20)	
	Access session, n (%)	Completed session, n (%)	Access session, n (%)	Completed session, n (%)
Week 1	20 (100)	18 (90)	20 (100)	17 (85)
Week 2	18 (90)	17 (85)	16 (80)	15 (75)
Week 3	16 (80)	15 (75)	17 (85)	13 (65)
Week 4	17 (85)	17 (85)	16 (80)	15 (75)
Week 5	17 (85)	15 (75)	14 (70)	14 (70)
Week 6	17 (80)	16 (80)	15 (75)	14 (70)

a ACT-BA: Acceptance and Commitment Therapy with Behavioral activation; average session completion rate=81.70%.

b TAU: Treatment-as-Usual; average session completion rate=73.33%.

### Missing Data Handling

This study had a total of 5.6% missing values. Little’s MCAR test indicated that the pattern of missing data followed the MCAR test (*χ*^2_10_^=0.000; *P*≥.99). However, due to substantial missingness (>5% missingness in posttest measures), MIs were used to preserve the statistical power and reduce bias, assuming that data were missing at random. Using SPSS, 10 imputed datasets were generated to calculate the pooled estimates. The results from the ITT analysis were compared with the PP analysis.

### Effectiveness of Intervention

#### Effectiveness of ACT-BA Intervention on Drinking-Related Outcomes

The ACT-BA intervention showed a significant group-by-time interaction effect on various drinking outcomes. Compared with the TAU group, participants in the intervention group showed a statistically significant reduction in drinking days (ITT: Group × time, *B*=−4.12, 95% CI −8.10 to −0.13, Wald *χ*²_4_=4.224, *P*=.04; PP: Group × time, *B*=−4.02, 95% CI −7.50 to −0.54, Wald *χ*²_1_=5.122, *P*=.02) and drinks per drinking day (ITT: Group × time, *B*=−1.56, 95% CI −3.06 to −0.07, Wald *χ*²_4_=4.298, *P*=.04; PP: Group × time, *B*=−1.31, 95% CI −2.60 to −0.02, Wald *χ*²_1_=3.956, *P*=.047) at postintervention. The ACT-BA intervention provided a moderate effect size on drinking days (Cohen *d*=−0.57) and a large effect size on drinks per drinking day (Cohen *d*=−1.89; Table 4). Although a statistically significant difference was not reached for CAD (ITT: Group × time, *B*=2.34, 95% CI −3.68 to 8.36, Wald *χ*²_4_=0.634, *P*=.43; PP: Group × time, *B*=2.36, 95% CI −2.45 to 7.17, Wald *χ*²_1_=.925, *P*=.34) and heavy drinking days (ITT: Group × time, *B*=−3.01, 95% CI −6.37 to −0.36, Wald *χ*²_4_=3.135, *P*=.08; PP: Group × time, *B*=−3.58, 95% CI −7.19 to 0.02, Wald *χ*²_1_=3.794, *P*=.05) at postintervention assessment (Table 5), the ACT-BA group showed improvement with small to moderate (Cohen *d*=0.32) and moderate to large (Cohen *d*=−0.71) effect sizes on these outcomes, respectively.

**Table 4.** Mean score and difference of the outcome variables for ACT-BA^a^ and TAU^b^ among ethnic minority young adults with AUD^c^ in Hong Kong across time points (pre and post; N=40): a pilot randomized controlled trial.

Outcomes	ACT-BA (n=20)			TAU (n=20)			Effect size
	Pretest, mean (SD)	Posttest, mean (SD)	Mean difference (SE)	Pretest, mean (SD)	Posttest, mean (SD)	Mean difference (SE)	Cohen *d*
AAES^d^							
PP^e^	44.45 (10.79)	58.69 (12.67)	14.24 (3.91)	49.55 (17.85)	51.11 (10.97)	1.56 (5.38)	0.83
ITT^f^	44.45 (10.79)	55.97 (17.39)	11.52 (4.58)	49.55 (17.85)	49.12 (13.95)	−0.43 (5.19)	0.81
AAQ-II^g^							
PP	24.45 (9.01)	14.31 (4.89)	−10.13 (2.51)	26.10 (10.76)	22.93 (5.33)	−3.17 (3.12)	−0.7
ITT	24.45 (9.01)	15.38 (6.31)	−9.07 (2.46)	26.10 (10.76)	23.44 (9.03)	−2.66 (3.15)	−0.65
RTCQ^h^							
PP	37.55 (8.26)	42.88 (6.55)	5.33 (2.53)	34.20 (10.90)	36.86 (12.62)	2.66 (4.05)	0.28
ITT	37.55 (8.26)	42.00 (7.69)	4.45 (2.52)	34.20 (10.90)	35.94 (13.64)	1.74 (3.90)	0.28
EDS^i^							
PP	19.50 (7.76)	11.31 (7.47)	−8.19 (2.56)	22.35 (9.16)	18.79 (10.34)	−3.56 (3.36)	−0.55
ITT	19.50 (7.76)	12.84 (9.03)	−6.66 (2.66)	22.35 (9.16)	19.73 (10.96)	−2.62 (3.30)	−0.48
DD^j^							
PP	9.35 (6.93)	5.56 (4.40)	−3.79 (1.99)	10.10 (7.62)	11.14 (7.81)	1.02 (2.68)	−0.66
ITT	9.35 (6.93)	5.98 (7.78)	−3.37 (2.14)	10.10 (7.62)	10.84 (9.44)	0.75 (2.71)	−0.57
DDD^k^							
PP	5.16 (0.75)	2.13 (1.45)	−3.03 (0.37)	5.03 (0.68)	3.32 (1.90)	−1.71 (0.46)	−1.84
ITT	5.16 (0.75)	2.45 (1.70)	−2.71 (0.42)	5.03 (0.68)	3.88 (2.72)	−1.15 (0.63)	−1.89
HDD^l^							
PP	6.55 (5.38)	2.88 (5.39)	−3.68 (1.81)	6.00 (2.64)	5.93 (6.31)	−0.71 (1.57)	−0.70
ITT	6.55 (5.38)	3.12 (5.99)	−3.43 (1.80)	6.00 (2.64)	5.57 (7.47)	−0.43 (1.77)	−0.71
CAD^m^							
PP	20.65 (6.93)	22.56 (7.30)	1.91 (2.38)	19.90 (7.62)	18.86 (7.81)	−1.04 (2.68)	0.41
ITT	20.65 (6.93)	22.52 (8.93)	1.87 (2.53)	19.90 (7.62)	19.43 (9.12)	−0.46 (2.65)	0.32

a ACT-BA: Acceptance and Commitment Therapy with Behavioral activation.

b TAU: Treatment-as-Usual.

c AUD: alcohol use disorder.

d AAES: Alcohol Abstinence Efficacy Scale.

e PP: per-protocol

f ITT: intention-to-treat.

g AAQ-II: Acceptance and Action Questionnaire—version II.

h RTCQ: Readiness to Change Questionnaires.

i EDS: Everyday Discrimination Scale.

j DD: drinks per day in the past 30 days.

k DDD: drinks per drinking day in the past 30 days.

l HDD: heavy drinking days in the past 30 days.

m CAD: cumulative abstinence durations in the past 30 days.

**Table 5.** Generalized Estimating Equations models with multiple imputation for the comparison of outcomes between the control and intervention groups of ethnic minority young adults with alcohol use disorder in Hong Kong across time points (pre and post; n=40): a pilot randomized controlled trial.

Outcomes and time points	ACT-BA^a^	TAU^b^	Group			Time			Group × time interaction		
	Mean (SE)	Mean (SE)	*B* (95% CI)	*P* value	Wald *χ*² (*df*)	*B* (95% CI)	*P* value	Wald *χ*² (*df*)	*B* (95% CI)	*P* value	Wald *χ*² (*df*)
AAES^c^											
GEE^d^											
Pre	44.45 (2.35)	49.55 (3.89)	1	≥.99	N/A^e^	1	N/A	N/A	1	≥.99	N/A
Post	58.73 (3.03)	51.11 (2.88)	−5.10 (−14.01 to 3.81)	.26	1.259 (1)	1.56 (−6.30 to 9.43)	.70	0.152 (1)	12.71 (2.79 to 22.64)	*.* .01	6.306 (1)
MI-GEE^f^											
Post	55.97 (3.83)	49.12 (3.26)	−0.43 (−9.69 to 8.82)	.93	1.259 (4)	−5.1 (−14.01 to 3.81)	.26	0.010 (4)	11.95 (0.10 to 23.81)	.05	3.960 (4)
RTCQ^g^										.01	
GEE											
Pre	37.55 (1.80)	34.20 (2.38)	1	≥.99		1	≥.99		1	≥.99	N/A
Post	43.49 (1.63)	37.65 (3.20)	3.35 (−2.49 to 9.19)	.26	1.264 (1)	3.45 (−5.47 to 12.36)	.45	0.574 (1)	2.50 (−7.79 to 12.79)	.23	0.227 (1)
MI-GEE											
Post	42.00 (1.68)	35.94 (2.99)	3.35 (−2.49 to 9.19)	.26	1.264 (4)	1.74 (−6.30 to 9.78)	.67	0.182 (4)	2.71 (−6.80 to 12.23)	.57	0.317 (4)
AAQ-II^h^											
GEE											
Pre	24.45 (1.96)	26.10 (2.35)	N/A	≥.99		1	≥.99		1	≥.99	N/A
Post	15.03 (1.25)	21.20 (1.84)	−1.65 (−7.65 to 4.34)	.59	0.291 (1)	−3.48 (−7.44 to −0.48)	.08	2.966 (1)	−6.93 (−11.95 to −1.92)	.001	7.333 (1)
MI-GEE											
Post	15.38 (1.38)	24.42 (1.62)	−1.65 (−7.65 to 4.35)	.59	0.291 (4)	−2.66 (−7.91 to 2.60)	.59	1.026 (4)	−6.41 (−12.77 to −0.06)	.048	3.960 (4)
EDS^i^											
GEE											
Pre	19.5 (1.69)	22.35 (2.00)	1	≥.99		1	≥.99	N/A	N/A	N/A	N/A
Post	11.6 (1.86)	19.72 (2.53)	−2.85 (−7.98 to 2.28)	.28	1.188 (1)	−2.62 (−7.04 to 1.79)	.22	1.360 (1)	−5.26 (−11.84 to 1.32)	.12	2.458 (1)
MI-GEE											
Post	12.84 (1.97)	19.73 (2.40)	−2.85 (−7.98 to 2.28)	.28	1.188 (4)	−2.62 (−7.28 to 2.04)	.27	1.229 (4)	−4.04 (−10.43 to 2.35)	.22	1.533 (4)
DD^j^											
GEE											
Pre	9.35 (1.51)	10.10 (1.66)	1	N/A	N/A	1	≥.99	N/A	N/A	N/A	N/A
Post	5.30 (0.74)	10.07 (1.64)	−0.75 (−5.15 to 3.65)	.74	0.112 (1)	−0.03 (−3.06 to 2.99)	.98	0.000 (1)	−4.02 (−7.50 to −0.54)	.02	5.122 (1)
MI-GEE											
Post	5.98 (1.45)	10.84 (2.08)	−0.75 (−5.15 to 3.65)	.74	0.112 (4)	0.75 (−3.21 to 4.70)	.70	0.153 (4)	−4.12 (−8.10 to −0.13)	.04	4.224 (4)
DDD^k^											
GEE											
Pre	5.16 (1.64)	5.03 (0.15)	1	≥.99	N/A	1	≥.99	N/A	1	≥.99	1
Post	2.14 (0.35)	3.33 (0.49)	0.13 (−0.31 to 0.56)	.56	0.339 (1)	−1.71 (−2.75 to −0.66)	.001	10.31 (1)	−1.31 (−2.60 to −0.02)	.05	3.956 (1)
MI-GEE											
Post	2.45 (0.37)	3.88 (0.59)	0.13 (−0.31 to 0.56)	.56	0.339 (4)	−1.15 (−2.43 to 0.12)	.08	3.336 (4)	−1.56 (−3.06 to −0.07)	.04	4.298 (4)
HDD^l^											
GEE											
Pre	6.55 (1.17)	6.00 (0.57)	1	≥.99	N/A	1	≥.99	N/A	1	≥.99	1
Post	2.77 (1.23)	5.69 (1.63)	0.55 (−2.01 to 3.11)	.67	0.177 (1)	−0.32 (−3.70 to 3.06)	.85	0.034 (1)	−3.58 (−7.19 to 0.02)	.05	3.794 (1)
MI-GEE											
Post	3.12 (1.32)	5.57 (1.65)	0.55 (−2.01 to 3.11)	.67	0.177 (4)	−0.43 (−3.90 to 3.05)	.80	0.064 (4)	−3.01 (−6.37 to −0.36)	.08	3.135 (4)
CAD^m^											
GEE											
Pre	20.65 (1.51)	19.9 (1.66)	N/A	N/A	N/A	N/A	N/A	N/A	N/A	N/A	N/A
Post	22.78 (1.68)	19.65 (1.71)	0.75 (−3.65 to 5.15)	.74	0.112 (1)	−0.251 (−3.14 to 2.63)	.86	0.030 (1)	2.36 (−2.45 to 7.17)	.34	0.925 (1)
MI-GEE											
Post	22.52 (1.96)	19.43 (2.00)	0.75 (−3.65 to 5.15)	.74	0.112 (4)	−0.47 (−4.16 to 3.23)	.80	0.067 (4)	2.34 (−3.68 to 8.36)	.43	0.634 (4)

a ACT-BA: Acceptance and Commitment Therapy with Behavioral activation.

b TAU: Treatment-as-Usual.

c AAES: Alcohol Abstinence Efficacy Scale.

d GEE: Generalized Estimating Equations.

e N/A: not applicable.

f MI-GEE: Multiple Imputation with Generalized Estimating Equation.

g RTCQ: Readiness to Change Questionnaires.

h AAQ-II: Acceptance and Action Questionnaire—version II.

i EDS: Everyday Discrimination Scale.

j DD: drinks per day in the past 30 days.

k DDD: drinks per drinking day in the past 30 days.

l HDD: heavy drinking days in the past 30 days.

m CAD: cumulative abstinence durations in the past 30 days.

#### Effectiveness of ACT-BA on AASE

A significant improvement in AASE was observed in the intervention group compared with those in the control group at postintervention. Participants who received the ACT-BA intervention showed a statistically significant increase in the mean AAES score from baseline to postintervention in both ITT and PP analyses (ITT: Group × time, *B*=11.95, 95% CI 0.10-23.81, Wald *χ*²_4_=3.960**,** *P*=.048; PP: Group × time, *B*=12.71, 95% CI 2.79-22.64, Wald *χ*²_1_=6.306, *P*=.01; Table 5). The effect size of the intervention on AASE was also large in both ITT (Cohen *d*=0.81) and PP (Cohen *d*=0.83) analyses (Table 4).

#### Effectiveness of ACT-BA on Psychological Flexibility

Participants in the intervention group revealed a statistically significant reduction in the mean score of Acceptance and Action Questionnaire—version II at postintervention in both analyses (ITT: Group × time, *B*=−6.41, 95% CI −12.77 to −0.06, Wald *χ*²_4_=3.960**,** *P*=.04; PP: Group × time, *B*=−6.93, 95% CI −11.95 to −1.92, Wald *χ*²_1_=7.333**,** *P*=.001). The effect size on psychological flexibility was moderate (ITT: Cohen *d*=−0.65; PP: Cohen *d*=−0.7; Table 4).

#### Effectiveness of ACT-BA on Readiness to Change

A mild improvement was observed in the mean Readiness to Change Questionnaire score in the intervention group in both analyses (ITT: Group × time, *B*=2.71, 95% CI −6.80 to 12.23, Wald *χ*²_4_=0.317, *P*=.57; PP: Group × time, *B*=2.50, 95% CI −7.79 to 12.79, Wald *χ*²_1_=0.227, *P*=.23; Table 5), with a small effect size in both ITT and PP analyses (Cohen *d*=0.28; Table 4).

#### Effectiveness of ACT-BA on Everyday Discrimination

As presented in Table 5, a greater improvement in the mean score of everyday discrimination was observed in the ACT-BA group compared with the TAU group at postintervention (ITT: Group × time, *B*=−4.04, 95% CI −10.43 to 2.35, Wald *χ*²_4_=1.533, *P*=.22; PP: Group × time, *B*=−5.26, 95% CI −11.84 to 1.32, Wald *χ*²_1_=2.458, *P*=.12) with a small effect size (ITT: Cohen *d*=−0.48; PP: Cohen *d*=−0.55).

### Participants’ Feelings, Experiences, and Confidence

Participants showed high satisfaction with the ACT-BA intervention for its guidance to manage drinking, living, thoughts, and emotions. For instance, in the semistructured interview, a participant said, “I find it very helpful for everyday living,” while others expressed its benefits as “I am happy as it helps people overcome drinking,” “a guide on how to live,” and a tool to “learn many things about drinking.” Another participant stated that the mindfulness exercise “go to nature, observe, and relax” reduced her stress. Participants also reported self-efficacy in engaging with the program due to its clear instructions, simple intervention content, perceived health benefits, and easy website navigation. For instance, a participant said, “The website is easy without assistance, I just followed the directions, 1 do not need assistance because I do understand the context. So, I did it on my own,” and “I’m confident in doing it. I just do it by myself.”

### Intervention Content, Delivery, and Duration

Most participants (n=11) expressed that the content was simple, with clear instructions. For instance, a participant described the content as “it’s well explained, easy to understand” and instructions as “it is easy because I tried to follow the instructions.” However, 2 of them noted challenges with unfamiliar terminology, particularly “need to search what words mean,” and task difficulty as “some parts are easy, some are hard.” All participants preferred the online delivery mode due to its flexibility and accessibility, and one said, “online is better because you can do it anywhere, anytime.” Despite the majority of participants enjoying the interventions, a few faced challenges related to the content, duration, and access to the intervention. Regarding duration, while most found the duration suitable, 2 participants suggested shortening it and said*, “*a little bit longer, but the interface is beautiful. Every content is important.” Additionally, one participant expressed difficulty in understanding some of the materials. In the semistructured interview, a participant said, “It’s quite difficult for me to understand some of the content.” Furthermore, 2 participants reported difficulties in accessing the intervention due to poor internet connection and stated that “sometimes there is an error when submitting, and another time there are errors in connection and login.”

### Perceived Usefulness of Interventions

As presented in Multimedia Appendix 1, the participants identified 5 themes regarding the benefits of the intervention. Participants reported increased awareness of their health and its consequences. For example, a participant said, “I got sick because of drinking alcohol… we are not getting younger.” Another participant also noted, “I read all the consequences...it helps me realize now what will happen if I keep on drinking...how would it affect me if I didn't control it.” By realizing the consequences, participants applied the BA strategies such as replacing drinking with alcohol-free activities. As reported by one participant, “I concentrate on other activities and don't drink as much as I can. I can do another thing instead of drinking because I know that drinking is not giving me anything more.” Some participants also avoided triggers by “limiting time in the triggering environment.” Besides, some participants learned to accept urges and negative emotions. One participant said, “I go to nature and just think and observe, and the switch can ease my mind, I can relax and reduce some of the stress.” Some also learned cognitive defusion and understood that “drinking too much can bring themselves out of their mind,” and such thinking helped them further detach from their urges. Overall, participants noted that this integrated intervention was a very good program and could be applied to their and others’ lives in the future. For instance, one of the participants said, “Oh, I think my feedback is that your program is very good. It can help other people...Honestly, I was a drinker before. Now, I realize that our body is not healthy because of alcohol. Even though I drink once a week, but this is not good. I suffered for almost 2 years, I got sick because of drinking alcohol.” Despite its usefulness, some participants reported difficulties in managing the withdrawal symptoms, for example, agitation and increased heart and respiratory rates, although they were motivated to quit alcohol use after the intervention.

## Discussion

### Principal Findings

This study was conducted among Hong Kong ethnic minority young adults with AUD, a population that often encounters barriers to health care access and has low treatment engagement and intervention adherence due to low-risk perception or poor health literacy [29,96]. To address this problem, we designed and evaluated a novel internet-based self-help program integrating ACT and BA among Hong Kong ethnic minority young adults with AUD.

This study provides preliminary evidence that our proposed intervention statistically significantly improved psychological flexibility, with a moderate effect size among ethnic minority young adults with AUD. This finding is consistent with previous studies on ACT for AUD [88,97]. However, our effect size on psychological flexibility is larger than that in previous studies, ranging from small to moderate [98]. The larger effect size might be due to the integration of ACT and BA [64,66]. Particularly, ACT emphasizes enhancing psychological flexibility in which participants learned different skills to allow difficult thoughts, feelings, and sensations, thus reducing experiential avoidance, which might trigger drinking as a coping behavior [50]. BA, as an additional component, further boosted the participants’ psychological flexibility by guiding them to identify a list of alternative value-congruent activities to replace drinking [99].

Our previous cross-sectional study and qualitative interviews identified that experiences of discrimination were a key factor leading to the development and maintenance of AUD among ethnic minority young adults [29]. Our quantitative findings showed a moderate effect on discrimination, revealing its potential efficacy in reducing the mean score of discrimination experiences, although a statistically significant mean difference was not reached between groups at postintervention. The insignificant difference between groups could be that our intervention did not aim to reduce the discrimination encountered by the participants [100]. Instead, the core focus of the intervention was to enhance the participants’ psychological flexibility to cope with the discrimination [50]. Another possible explanation for the insignificant difference is that the sample size of this study was small, resulting in a type 2 error in the analysis [101].

The ACT-BA intervention successfully enhanced participants’ AASE in the intervention group compared with the TAU group, with a large effect size at postintervention. AASE is defined as personal confidence in one’s own ability to abstain from alcohol [102]. Bandura [103] (1977) pointed out that self-efficacy is an important construct in determining whether a person will make attempts and take actions for behavioral changes. Although our intervention did not contain any strategy that could directly improve self-efficacy in alcohol abstinence among our participants, it can be indirectly and gradually built up in our intervention through participants’ successful experience in alcohol reduction when they tried to cut down on drinking [104]. Based on this preliminary finding, we expect that the intervention effect on AASE would be stronger at a later time because of more successful experience in alcohol reduction [105]. This postulation shall be further confirmed by a full-scale RCT with longer follow-up.

In this study, the ACT-BA was demonstrated to have statistically significant effects on some drinking outcomes. Particularly, compared with the TAU group, participants in the intervention group reported an average reduction of 4.12 drinking days per month and 1.15 drinks per drinking day at postintervention, with −0.57 and −1.89 effect sizes, respectively. These reductions are perhaps a reflection of the synergetic efficacy of the integrated intervention on the participants’ psychological flexibility, which emphasized noticing negative and unfavorable feelings without judgment, while adapting alternative behaviors to replace drinking in view of pursuing life goals [106]. Notwithstanding a statistically significant mean difference on the number of drinking days and number of drinks per drinking day, we did not find any statistically significant difference on the number of heavy drinking days and cumulative alcohol abstinence, but still the intervention group showed improvement on those outcomes. The insignificant mean difference for these 2 outcomes was likely because complete abstinence from alcohol could trigger withdrawal symptoms, which were not heavily emphasized in our intervention, and these withdrawal symptoms were hard to overcome by only improving their psychological flexibility and self-efficacy in alcohol abstinence [107,108]. This explanation is also supported by our qualitative findings that some participants in the experimental group reported difficulties in managing withdrawal symptoms, for example, agitation and increased heart and respiratory rates, although they were motivated to quit alcohol after the intervention. In response to this issue, this intervention can be strengthened, on one hand, by increasing the content about the management of withdrawal symptoms and, on the other hand, by referring the participants who may require medications for withdrawal symptoms to health care professionals alongside the intervention [109]. Another possible explanation for the insignificant findings was that reduction in heavy drinking and alcohol abstinence are more distal outcomes, which could only be captured and detected by a longer follow-up, which is 6 months, a widely accepted international standard for evaluation of behavioral changes [110]. Thus, a follow-up duration of 6 months or more is recommended for future full-scale RCTs.

Although no significant difference was observed between groups regarding readiness to change, mainly due to the limited power of the study, the intervention group showed improvement in such outcome at postintervention in ITT analysis (*B*=2.71). Again, this was likely because our proposed intervention focused on enhancing psychological flexibility and value-driven action skills rather than the shift in stages of readiness to change [77]. Additionally, the nonsignificant group-by-time interaction could be partly attributed to a ceiling effect, as most participants reported a high score of readiness to change at baseline, resulting from the convenience sampling technique [111].

The study revealed that the ACT-BA program was feasible for ethnic minority young adults with AUD, evidenced by a high intervention completion of 80% and a low attrition rate of 15%. This completion rate is higher than those reported in existing studies on various interventions, including ACT for AUD [57,98]. The variation could be attributable to 2 innovations of this study. First, the incorporation of BA strategies into ACT interventions may enhance engagement through value-aligned activity substitution. Second, when compared with the traditional face-to-face method to deliver ACT, our intervention was delivered using an online platform with no fixed schedule, allowing the participants to access the intervention at their convenience throughout the week, minimizing work disruptions [112]. While there was variation in time between sessions (2 median intervals of 7 days; IQR days), ethnic minority young adults confirmed that a 6-week duration was appropriate, consistent with existing literature [56].

Our postintervention semistructured interview revealed an overall high satisfaction among the participants in the experimental group, with all endorsing the intervention as effective in guiding them to reduce alcohol consumption, improve emotional regulation, and enhance life management. The participants particularly highlighted that the intervention has clear instructions, simplified content, and easy website navigation, enhancing their interest in engaging with the intervention. In their interview, most participants described the intervention sessions as “simple, well explained, and easy to understand.” All participants preferred the online delivery format due to its flexibility and accessibility [112]. Importantly, the participants reported various degrees in behavior change in their alcohol use and expressed intent to practice and sustain the learned intervention skills in the long term. These positive responses from the participants provide compelling evidence to support the feasibility of the ACT-BA, apart from the feasibility indicators.

### Limitations

This RCT has several limitations. First, the small sample size may reduce the statistical power, potentially affecting the true effect size of the interventions. Second, the self-help intervention limited direct measurement of the participants’ engagement with exercises, metaphors, and practices of each module. Nevertheless, engagement was assessed indirectly through online tracking of the module completion time and daily exercise logs. Third, the preliminary effectiveness was assessed immediately postintervention. Although psychosocial interventions usually bring behavioral changes over time following the intervention, the absence of follow-up assessment may limit the evaluation of the long-term effect of the ACT-BA. Fourth, this study relied solely on participants’ self-reported data rather than objective assessments, which might introduce bias and potentially lead to an over- or underestimation of the effect size of the outcome variables. Fifth, although the ACT-BA showed significant effects across multiple outcomes, how the effects varied across different ethnic minority groups was not examined. Sixth, although almost all ethnic minority participants were able to speak English, excluding individuals who do not speak English may have introduced selection bias, potentially limiting the generalizability of findings to all ethnic minority groups.

### Future Implications

This study addresses the literature gaps by providing evidence on the preliminary effectiveness and feasibility of the ACT-BA on different drinking-related outcomes among ethnic minorities with AUD. Therefore, a key implication for future study was to conduct a fully powered RCT to further examine the effectiveness of such intervention, with longer follow-up to capture the long-term effect on different drinking outcomes and incorporating objective measures to assess the drinking outcomes to minimize the social desirability bias. Given that the intervention can be self-administered in an online platform with only minimal involvement from interventionalists in a flexible schedule for working groups and at a low cost, it is expected that this intervention, if proven to be effective in a large-scale similar RCT, can be further delivered to a large group of ethnic minority young adults in the community.

### Conclusions

This study presents an innovative integrated ACT and BA intervention, which has been delivered through an internet-based self-help format for ethnic minority young adults with AUD. Unlike the existing evidence that mainly focuses on a single intervention, either ACT or BA, this study integrated those 2 interventions to enhance improvement in abstinence. The findings contribute to the field by providing preliminary evidence for both the feasibility and the potential efficacy of this novel, self-help, flexible, and low-intensity integrated intervention. Feasibility was supported by the high completion rate and low attrition rate, while preliminary efficacy was reflected in reductions in drinks per drinking day and drinking days, alongside improvements in AASE and psychological flexibility among ethnic minority young adults with AUD. The main implication of this study is that this intervention would serve as a key treatment option for ethnic minorities with AUD who encounter challenges in face-to-face therapy. Given these promising findings, the effectiveness of the ACT-BA should be further confirmed in a fully powered RCT before its implementation for this population group in the community.

## Supplementary material

10.2196/83896 Multimedia Appendix 1 Themes, codes, sample quotes, and descriptions for the qualitative findings. 1, 2

10.2196/83896 Checklist 1 CONSORT-eHEALTH checklist (V 1.6.1).

10.2196/83896 Checklist 2 CONSORT 2025 checklist for reporting a randomized trial.

## Abbreviations

AASE: alcohol abstinence self-efficacy
ACT: acceptance and commitment therapy
ACT-BA: Acceptance and Commitment Therapy with Behavioral Activation
AUD: alcohol use disorder
BA: behavioral activation
CAD: cumulative abstinence duration
CONSORT: Consolidated Standards of Reporting Trials
COREQ: Consolidated Criteria for Reporting Qualitative Research
*DSM-5*: *Diagnostic and Statistical Manual of Mental Disorders* (Fifth Edition)
GEE: Generalized Estimating Equations
ITT: intention-to-treat
MCAR: Missing Completely at Random
MI: multiple imputation
PP: per-protocol
RA: research assistant
RCT: randomized controlled trial
SUD: substance use disorder
TAU: Treatment-as-Usual

## References

[1] (2022). Understanding alcohol use disorder. National Institute on Alcohol Abuse and Alcoholism.

[2] (2022). Substance use in adolescents. MSD MANUAL.

[3] Naar S (2023). The transition from adolescence to adulthood. Psychology Today.

[4] (2025). What increases the risk for alcohol use disorder?. National Institute on Alcohol Abuse and Alcoholism.

[5] Dawson DA, Goldstein RB, Chou SP, Ruan WJ, Grant BF (2008). Age at first drink and the first incidence of adult-onset DSM-IV alcohol use disorders. Alcohol Clin Exp Res.

[6] Squeglia LM (2020). Alcohol and the developing adolescent brain. World Psychiatry.

[7] (2022). The global action for measurement of adolescent health (GAMA). World Health Organization.

[8] Sullivan M, Risler E (2002). Understanding college alcohol abuse and academic performance: selecting appropriate intervention strategies. J of College Counseling.

[9] Castaño-Perez GA, Calderon-Vallejo GA (2014). Problems associated with alcohol consumption by university students. Rev Lat Am Enfermagem.

[10] Oluwafemi A (2020). Alcohol consumption as anticipator of academic performance among undergraduate students (a case study of Ondo State University of Science and Technology Okitipupa, Ondo State Nigeria). IFE PsychologIA Int J.

[11] Sindelar HA, Barnett NP, Spirito A (2004). Adolescent alcohol use and injury. A summary and critical review of the literature. Minerva Pediatr.

[12] Hingson RW, Zha W (2009). Age of drinking onset, alcohol use disorders, frequent heavy drinking, and unintentionally injuring oneself and others after drinking. Pediatrics.

[13] Callaghan RC, Gatley JM, Veldhuizen S, Lev-Ran S, Mann R, Asbridge M (2013). Alcohol- or drug-use disorders and motor vehicle accident mortality: a retrospective cohort study. Accident Analysis & Prevention.

[14] Holcomb RL (1938). Alcohol in relation to traffic accidents. JAMA.

[15] (2022). Alcohol, fact sheet. World Health Organization.

[16] Cheung YW (1993). Ethnic identification and alcohol use among Canadian-born and foreign-born high school students in Toronto. Int J Addict.

[17] Edwards RW, Thurman PJ, Beauvais F (1995). Patterns of alcohol use among ethnic minority adolescent women. Recent Dev Alcohol.

[18] Statistics CfBH, Quality (2021). Racial/ethnic differences in substance use, substance use disorders, and substance use treatment utilization among people aged 12 or older (2015-2019).

[19] (2021). The demographics: ethnic groups. Relation Units.

[20] (2022). Discrimination and mental health—a guide for young people. Cool Minds.

[21] Dominic L, Michelle L (2011). Being young and a minority: a study of the challenges encountered by young ethnic minorities in Hong Kong. J Youth Stud.

[22] Rohde P, Lewinsohn PM, Kahler CW, Seeley JR, Brown RA (2001). Natural course of alcohol use disorders from adolescence to young adulthood. J Am Acad Child Adolesc Psychiatry.

[23] Chou T, Asnaani A, Hofmann SG (2012). Perception of racial discrimination and psychopathology across three U.S. ethnic minority groups. Cultur Divers Ethnic Minor Psychol.

[24] Demombynes G (2013). Why is ethnic minority poverty persistent in Vietnam. https://blogs%20worldbank%20org/eastasiapacific/why-ethnic-minority-poverty-persistent-vietnam.

[25] Liu MA, Prestigiacomo CJ, Karim MFA, Ashburn-Nardo L, Cyders MA (2024). Psychological outcomes and culturally relevant moderators associated with events of discrimination among Asian American adults. Cultur Divers Ethnic Minor Psychol.

[26] Vaeth PAC, Wang-Schweig M, Caetano R (2017). Drinking, alcohol use disorder, and treatment access and utilization among U.S. racial/ethnic groups. Alcohol Clin Exp Res.

[27] Vandan N, Wong JYH, Lee JJJ, Yip PSF, Fong DYT (2020). Challenges of healthcare professionals in providing care to South Asian ethnic minority patients in Hong Kong: a qualitative study. Health Soc Care Community.

[28] (2021). Research on social inclusion: employment situations of ethnic minority in Hong Kong. TREATS.

[29] Belay GM, Wah MY, Lam KKW (2025). Reasons and impacts of alcohol use disorder among ethnic minority young adults: a descriptive phenomenological study. Front Public Health.

[30] Bernstein J, Heeren T, Edward E (2010). A brief motivational interview in a pediatric emergency department, plus 10-day telephone follow-up, increases attempts to quit drinking among youth and young adults who screen positive for problematic drinking. Acad Emerg Med.

[31] Borsari B, Short EE, Mastroleo NR (2014). Phone-delivered brief motivational interventions for mandated college students delivered during the summer months. J Subst Abuse Treat.

[32] Brown RA, Abrantes AM, Minami H (2015). Motivational interviewing to reduce substance use in adolescents with psychiatric comorbidity. J Subst Abuse Treat.

[33] Newton AS, Dow N, Dong K (2017). A randomised controlled pilot trial evaluating feasibility and acceptability of a computer-based tool to identify and reduce harmful and hazardous drinking among adolescents with alcohol-related presentations in Canadian pediatric emergency departments. BMJ Open.

[34] Segatto ML, Andreoni S, de Souza e Silva R, Diehl A, Pinsky I (2011). Brief motivational interview and educational brochure in emergency room settings for adolescents and young adults with alcohol-related problems: a randomized single-blind clinical trial. Braz J Psychiatry.

[35] Wagner EF, Hospital MM, Graziano JN, Morris SL, Gil AG (2014). A randomized controlled trial of guided self-change with minority adolescents. J Consult Clin Psychol.

[36] Slesnick N, Prestopnik JL (2009). Comparison of family therapy outcome with alcohol-abusing, runaway adolescents. J Marital Fam Ther.

[37] Arnaud N, Diestelkamp S, Wartberg L, Sack PM, Daubmann A, Thomasius R (2017). Short- to midterm effectiveness of a brief motivational intervention to reduce alcohol use and related problems for alcohol intoxicated children and adolescents in pediatric emergency departments: a randomized controlled trial. Acad Emerg Med.

[38] Martín-Pérez C, Navas JF, Perales JC (2019). Brief group-delivered motivational interviewing is equally effective as brief group-delivered cognitive-behavioral therapy at reducing alcohol use in risky college drinkers. PLoS One.

[39] Spirito A, Monti PM, Barnett NP (2004). A randomized clinical trial of a brief motivational intervention for alcohol-positive adolescents treated in an emergency department. J Pediatr.

[40] Latimer WW, Winters KC, D’Zurilla T, Nichols M (2003). Integrated family and cognitive-behavioral therapy for adolescent substance abusers: a stage I efficacy study. Drug Alcohol Depend.

[41] Kaminer Y, Burleson JA, Burke RH (2008). Efficacy of outpatient aftercare for adolescents with alcohol use disorders: a randomized controlled study. J Am Acad Child Adolesc Psychiatry.

[42] Murray LK, Kane JC, Glass N (2020). Effectiveness of the Common Elements Treatment Approach (CETA) in reducing intimate partner violence and hazardous alcohol use in Zambia (VATU): a randomized controlled trial. PLoS Med.

[43] Belay GM, Mak YW, Wong FKY (2024). Psychosocial treatment options for adolescents and young adults with alcohol use disorder: systematic review and meta-analysis. Front Public Health.

[44] Perkins AM, Meiser-Stedman R, Spaul SW, Bowers G, Perkins AG, Pass L (2023). The effectiveness of third wave cognitive behavioural therapies for children and adolescents: a systematic review and meta-analysis. Br J Clin Psychol.

[45] Kahl KG, Winter L, Schweiger U (2012). The third wave of cognitive behavioural therapies: what is new and what is effective?. Curr Opin Psychiatry.

[46] Dela Cruz GA, Johnstone S, Kim HS, Castle DJ (2023). Review of third-wave therapies for substance use disorders in people of color and collectivist cultures: current evidence and future directions. Psychol Addict Behav.

[47] Vujanovic AA, Meyer TD, Heads AM, Stotts AL, Villarreal YR, Schmitz JM (2017). Cognitive-behavioral therapies for depression and substance use disorders: an overview of traditional, third-wave, and transdiagnostic approaches. Am J Drug Alcohol Abuse.

[48] Hayes SC (2004). Acceptance and commitment therapy, relational frame theory, and the third wave of behavioral and cognitive therapies. Behav Ther.

[49] Stotts AL, Northrup TF (2015). The promise of third-wave behavioral therapies in the treatment of substance use disorders. Curr Opin Psychol.

[50] Hayes SC, Luoma JB, Bond FW, Masuda A, Lillis J (2006). Acceptance and commitment therapy: model, processes and outcomes. Behav Res Ther.

[51] Hayes SC, Strosahl KD (2004). A Practical Guide to Acceptance and Commitment Therapy.

[52] Forman EM, Juarascio AS, Martin LM, Herbert JD (2014). Acceptance and commitment therapy (ACT). The Encyclopedia of Clinical Psychology.

[53] Sevinçok D, Memiş ÇÖ, Çakaloz B (2018). Acceptance and commitment therapy experience in an adolescent patient with social anxiety disorder, panic disorder, and major depression. Psychiatry Clin Psychopharmacol.

[54] Lee EB, An W, Levin ME, Twohig MP (2015). An initial meta-analysis of Acceptance and Commitment Therapy for treating substance use disorders. Drug Alcohol Depend.

[55] Osaji J, Ojimba C, Ahmed S (2020). The use of acceptance and commitment therapy in substance use disorders: a review of literature. J Clin Med Res.

[56] Levin ME, Haeger JA, Pierce BG, Twohig MP (2017). Web-based acceptance and commitment therapy for mental health problems in college students: a randomized controlled trial. Behav Modif.

[57] Khandelwal N, Das K, Sharma R, Ghosh A (2024). Testing the waters: a pilot trial of acceptance and commitment therapy (ACT) for alcohol use disorder. Indian J Psychiatry.

[58] Brown M, Hooper N, James P, Scott D, Bodger O, John A (2020). A web-delivered acceptance and commitment therapy intervention with email reminders to enhance subjective well-being and encourage engagement with lifestyle behavior change in health care staff: randomized cluster feasibility stud. JMIR Form Res.

[59] Clark KR (2018). Learning theories: Behaviorism. Radiol Technol.

[60] Martell CR, Dimidjian S, Herman-Dunn R, Barlow DH Clinical Handbook of Psychological Disorders: A Step-by-Step Treatment Manual.

[61] Kanter JW, Manos RC, Bowe WM, Baruch DE, Busch AM, Rusch LC (2010). What is behavioral activation? A review of the empirical literature. Clin Psychol Rev.

[62] Santos MM, Puspitasari AJ, Nagy GA, Kanter JW, Wenzel A (2021). Handbook of Cognitive Behavioral Therapy: Overview and Approaches.

[63] Fernández-Rodríguez C, Coto-Lesmes R, Martínez-Loredo V, González-Fernández S, Cuesta M (2023). Is activation the active ingredient of transdiagnostic therapies? A randomized clinical trial of behavioral activation, acceptance and commitment therapy, and transdiagnostic cognitive-behavioral therapy for emotional disorders. Behav Modif.

[64] Gaudiano BA, Nowlan K, Brown LA, Epstein-Lubow G, Miller IW (2013). An open trial of a new acceptance-based behavioral treatment for major depression with psychotic features. Behav Modif.

[65] Daughters SB, Magidson JF, Lejuez CW, Chen Y (2016). LETS ACT: a behavioral activation treatment for substance use and depression. Adv Dual Diagn.

[66] Carlbring P, Hägglund M, Luthström A (2013). Internet-based behavioral activation and acceptance-based treatment for depression: a randomized controlled trial. J Affect Disord.

[67] Moock J (2014). Support from the internet for individuals with mental disorders: advantages and disadvantages of e-mental health service delivery. Front Public Health.

[68] Taylor CB, Luce KH (2003). Computer- and internet-based psychotherapy interventions. Curr Dir Psychol Sci.

[69] Andersson G, Titov N (2014). Advantages and limitations of Internet-based interventions for common mental disorders. World Psychiatry.

[70] Thomas N, McLeod B, Jones N, Abbott JA (2015). Developing Internet interventions to target the individual impact of stigma in health conditions. Internet Interv.

[71] Cunningham JA, Wild TC, Cordingley J, van Mierlo T, Humphreys K (2009). A randomized controlled trial of an internet-based intervention for alcohol abusers. Addiction.

[72] Lappalainen P, Granlund A, Siltanen S (2014). ACT internet-based vs face-to-face? A randomized controlled trial of two ways to deliver Acceptance and Commitment Therapy for depressive symptoms: an 18-month follow-up. Behav Res Ther.

[73] Blankers M, Nabitz U, Smit F, Koeter MWJ, Schippers GM (2012). Economic evaluation of internet-based interventions for harmful alcohol use alongside a pragmatic randomized controlled trial. J Med Internet Res.

[74] Pots WTM, Fledderus M, Meulenbeek PAM, ten Klooster PM, Schreurs KMG, Bohlmeijer ET (2016). Acceptance and commitment therapy as a web-based intervention for depressive symptoms: randomised controlled trial. Br J Psychiatry.

[75] Zettle R (2007). ACT for Depression: A Clinician’s Guide to Using Acceptance and Commitment Therapy in Treating Depression.

[76] Hayes SC, Strosahl KD, Wilson KG (1999). Acceptance and Commitment Therapy: The Process and Practice of Mindful Change.

[77] Hayes SC, Pierson H (1999). Encyclopedia of Cognitive Behavior Therapy.

[78] Vuchinich RE, Tucker JA (1988). Contributions from behavioral theories of choice to an analysis of alcohol abuse. J Abnorm Psychol.

[79] Daughters SB, Magidson JF, Anand D, Seitz-Brown CJ, Chen Y, Baker S (2018). The effect of a behavioral activation treatment for substance use on post-treatment abstinence: a randomized controlled trial. Addiction.

[80] (2022). Population, total—Hong Kong SAR. World Bank.

[81] (2021). Alcohol use disorder: a comparison between DSM–IV and DSM–5 on alcohol abuse or alcoholism. National Institute on Alcohol Abuse and Alcoholism.

[82] Vespa J (2017). The changing economics and demographics of young adulthood: 1975-2016. https://hispanicad.com/wp-content/uploads/2017/04/p20-579.pdf.

[83] Liu T, Gietel-Basten S (2019). The demography of drug abuse in Hong Kong. China J Soc Work.

[84] Julious SA (2005). Sample size of 12 per group rule of thumb for a pilot study. Pharm Stat.

[85] Johns EK, Kokokyi S, Neufeld D, Krysanski V, Meek BP (2024). Taking ACTion for anxiety and depression: a pilot study of a brief virtual acceptance and commitment therapy group in primary care. J Contextual Behav Sci.

[86] Petersen CL, Zettle RD (2009). Treating inpatients with comorbid depression and alcohol use disorders: a comparison of acceptance and commitment therapy versus treatment as usual. Psychol Rec.

[87] Thekiso TB, Murphy P, Milnes J, Lambe K, Curtin A, Farren CK (2015). Acceptance and commitment therapy in the treatment of alcohol use disorder and comorbid affective disorder: a pilot matched control trial. Behav Ther.

[88] Weststrate TR, Briggs CA, Miller A, Shuster AE, Gaynor ST (2023). Brief acceptance and commitment therapy added to medication management during acute alcohol detoxification: A pilot randomized controlled effectiveness trial. J Contextual Behav Sci.

[89] (2025). Understanding alcohol drinking patterns. National Institute on Alcohol Abuse and Alcoholism.

[90] Heather N, Rollnick S (1993). Readiness to Change Questionnaire: user’s manual. https://ndarc.med.unsw.edu.au/sites/default/files/ndarc/resources/TR.019.pdf.

[91] Bond FW, Hayes SC, Baer RA (2011). Preliminary psychometric properties of the Acceptance and Action Questionnaire-II: a revised measure of psychological inflexibility and experiential avoidance. Behav Ther.

[92] Belay GM, Wai KLK, Mak YW (2026). Factors associated with alcohol use disorder among young adults from ethnic minorities: a cross-sectional study. BMC Psychol.

[93] Sternthal MJ, Slopen N, Williams DR (2011). Racial disparities in health: how much does stress really matter?. Du Bois Rev.

[94] Hopewell S, Chan AW, Collins GS (2025). CONSORT 2025 statement: updated guideline for reporting randomised trials. Lancet.

[95] Tong A, Sainsbury P, Craig J (2007). Consolidated Criteria for Reporting Qualitative Research (COREQ): a 32-item checklist for interviews and focus groups. Int J Qual Health Care.

[96] Vandan N, Wong JYH, Fong DYT (2019). Accessing health care: experiences of South Asian ethnic minority women in Hong Kong. Nurs Health Sci.

[97] Svanberg G, Munck I, Levander M (2017). Acceptance and commitment therapy for clients institutionalized for severe substance-use disorder: a pilot study. Subst Abuse Rehabil.

[98] Meyer EC, Walser R, Hermann B (2018). Acceptance and commitment therapy for co-occurring posttraumatic stress disorder and alcohol use disorders in veterans: pilot treatment outcomes. J Trauma Stress.

[99] Ghodrati S, Vaziri Nekoo R (2018). The effectiveness of behavioral activation (BA) on psychological well-being and psychological flexibility in female students. J Med Counc Iran.

[100] Harris R (2019). ACT made simple: an easy-to-read primer on acceptance and commitment therapy. https://www.actmindfully.com.au/upimages/ACT_Made_Simple_Introduction_and_first_two_chapters.pdf.

[101] Akobeng AK (2016). Understanding type I and type II errors, statistical power and sample size. Acta Paediatr.

[102] DiClemente CC, Carbonari JP, Montgomery RP, Hughes SO (1994). The Alcohol Abstinence Self-Efficacy Scale. J Stud Alcohol.

[103] Bandura A (1977). Self-efficacy: toward a unifying theory of behavioral change. Psychol Rev.

[104] Kadden RM, Litt MD (2011). The role of self-efficacy in the treatment of substance use disorders. Addict Behav.

[105] Rollnick S, Heather N (1982). The application of Bandura’s self-efficacy theory to abstinence-oriented alcoholism treatment. Addict Behav.

[106] Reynolds EK, Macpherson L, Tull MT, Baruch DE, Lejuez CW (2011). Integration of the brief behavioral activation treatment for depression (BATD) into a college orientation program: depression and alcohol outcomes. J Couns Psychol.

[107] Hall W, Zador D (1997). The alcohol withdrawal syndrome. Lancet.

[108] Becker HC (2008). Alcohol dependence, withdrawal, and relapse. Alcohol Res Health.

[109] Kattimani S, Bharadwaj B (2013). Clinical management of alcohol withdrawal: a systematic review. Ind Psychiatry J.

[110] Wayne W (2022). The transtheoretical model (stages of change). Encyclopedia.

[111] Andrade C (2021). The ceiling effect, the floor effect, and the importance of active and placebo control arms in randomized controlled trials of an investigational drug. Indian J Psychol Med.

[112] Rafieifar M, Hanbidge AS, Lorenzini SB, Macgowan MJ (2025). Comparative efficacy of online vs. face-to-face group interventions: a systematic review. Res Soc Work Pract.

