# Supplementary material for: Effect of Integrated Internet-Based Acceptance and Commitment Therapy and Behavioral Activation Among Ethnic Minority Young Adults With Alcohol Use Disorder in Hong Kong: Pilot Randomized Controlled Trial
Source: J Med Internet Res. 2026 May 21;28:e83896. doi: 10.2196/83896 (PMC13193706; doi:10.2196/83896)
Supplement: Multimedia Appendix 1 [file jmir-v28-e83896-s001.docx]

Multimedia Appendix 1

Themes, codes, sample quotes, and descriptions for the qualitative findings.

| **Themes** | **Codes** | **Sample quotes** | **Descriptions** |
| --- | --- | --- | --- |
| **Intervention content, delivery, duration, and confidence** | |  | Participants expressed the convenience and flexibility of the online method: For instance, the quotes include “online is ok, no time for face to face, and “you can do it anywhere, anytime” with smart phone, which allows participants to engage in the program at their schedule without time and location constraints. Self-help delivery allows users to understand, “more time to understand and analyze it.” |
| Program flexibility and accessibility | Convenience | Online is ok, no time for face-to-face |  |
|  |  | It's better when you give online, because you can do it anywhere, anytime |  |
|  |  | I think it's better online because anytime you can read, you know. |  |
|  |  | I think it's online because anytime we can open it, because I can touch my phone and see it. |  |
|  |  | it's better online so that you can see it, because online, maybe you may have more time to understand the context and understand it, think about it, and then analyse it |  |
|  |  | I think, uh, providing online only because sometimes I'm quite busy. I'm holding my phone. |  |
| Perceived duration suitability | Appropriate duration | The duration it's OK. It was great, it helps me with so many things as sessions like the one observing nature and going to places, and just thinking and observing | Most participants found the intervention's duration acceptable. Despite some stating the suitability, others faced time constraints: “I am busy working.” |
|  |  | It's good, suitable with me |  |
|  |  | The timing is OK, but the problem is…I'm busy working |  |
| Self-efficacy and engagement | Confident | I'm confident in doing it. I just do it by myself | Participants reported high confidence in engagement. Self-efficacy, clear instructions, and simple intervention content enhanced independence without assistance. |
|  | Confident | I have the confidence by myself because I need to be healthy |  |
|  | Confident | Yeah, I'm very confident. I only do it by myself |  |
|  | Engagement | I did it on my own. I did not need assistance. |  |
|  | Independent use | The website is easy without any assistance. I just followed the directions, and 1 do not need assistance because I do understand the context. So, I did it on my own |  |
|  | Easy | That makes everything understandable. I'm very comfortable. |  |
|  | Engagement | I did it because I need to become healthy physically, mentally, and emotionally. |  |

(Continued…): Themes, codes, sample quotes, and descriptions for the qualitative findings.

| **Themes** | **Codes** | **Sample quotes** | **Descriptions** |
| --- | --- | --- | --- |
| **Instruction clarity** | Simple  guidance | It's simple to understand. I read the directions, followed them, and applied them to myself. | Participants reported that clear instructions, explanatory videos, and practical examples made the self-help online intervention simple and understandable. |
|  |  | The videos…. well explained, I understand when I watch |  |
|  |  | They always give specific examples so you can understand what we are doing. I followed an example |  |
|  |  | It is easy for me because I tried to follow the instructions. |  |
| **Simplicity of content** | Simple | It's simple to understand | The use of straightforward language along with audio and/or video, detailed actionable examples, and instructions, “easy to understand”, “well explained”, and “easy to follow instructions” ensures the simplicity of content for further iterations. |
|  | Easy | It is better performed and easier to understand |  |
|  | Simple | It is better performed and easier to understand |  |
|  | Easy navigation | It's very easy to follow…not difficult to answer |  |
|  | Clear | It's well explained. I understand that |  |
|  | Easy | It is not quite difficult for me to understand that. So, it's fine and it's good. |  |
|  | Simple | It's simple to understand. I read the directions, followed them, and applied them to myself. |  |
| **Challenges in content, time, and connection** | Unfamiliar terms | Some words…you need to look up what they mean | While most participants suggested the intervention was simple with suitable durations, a few participants faced minor challenges, such as unfamiliar terminology, task difficulty, and technical issues, including submission errors and unstable internet connectivity. Two participants stated that the program duration is a bit longer and needs to be shortened for further intervention. |
|  | Difficult task | Some parts are easy, some are hard… |  |
|  | Difficult task | Some of the contents of the modules need assistance because it's. It's quite difficult for me to understand some of the content. |  |
|  | Need for brevity | A little bit long…. should be short |  |
|  | Need for brevity | I take a little bit longer |  |
|  | Technical error | Sometimes error when I submit. but overall, it's good and nice |  |
|  | Internet problem | Sometimes have errors on the Internet |  |
|  | Withdrawal symptom | difficulties in managing withdrawal symptoms, e.g., agitation and increased heart and respiratory rates |  |

Themes, codes, sample quotes, and descriptions for the qualitative findings.

| **Themes** | | **Codes** | **Sample quotes** | **Descriptions** |
| --- | --- | --- | --- | --- |
| **Perceived usefulness of intervention** | |  |  |  |
| **Health awareness and consequences** | Consequence’s recognition | Yeah, intervention is beneficial. I see the difference between I'm not drinking and drinking, not only in my health, but also in my body | Participants benefited from the interventions. They detached from unhelpful thoughts about alcohol, “I realize that what happens if I keep drinking…how would it affect me if I do not control it,” accepting negative emotions with mindfulness exercise by nature observation instead of avoiding urges. go to nature, observe, and relax… helps me manage emotions without drinking” |  |
|  | Consequence recognition | I suffered for almost 2 years; I got sick because of drinking alcohol… we are not getting younger |  |  |
|  | Notice Consequence | Yeah, it's useful to me as I read all the consequences…it helps me realize now what will happen if I keep on drinking… how would it affect me if I didn't control it? |  |  |
|  | Risk awareness | It is useful, like I mean, I can share with another soul, so that drinking alcohol is not good for our health |  |  |
|  |  | It's like a guide to me on how to live. How will it affect me if I keep on drinking? |  |  |
| **Behavioral change strategies** | Trigger avoidance | Oh, yeah…I limit my time of coming to chunking… It's a good thing. It's a good program because it helped me. You know, it's not like everyday drinking |  |  |
|  | Track consumption | It was useful for tracking the drinking things, the drinking stuff. It also helps to quit or stop drinking alcohol |  |  |
|  | Activity scheduling | I concentrate on other activities and don't drink as much as I used to. I can do another thing instead of drinking because I know that drinking does not give me anything more. |  |  |
|  | Activity scheduling | The benefit is that it helps me think about alcohol. Having activities, your daily game, so that you can lessen the drinking, which helps |  |  |

(Continued…): Themes, codes, sample quotes, and descriptions for the qualitative findings.

| **Themes** | **Codes** | **Sample quotes** |  |
| --- | --- | --- | --- |
| **Cognitive and Emotional Regulation** | Cognitive defusion | Yes, for me, keep this to apply it in the future…. If you drink, don’t drink much because that thing can bring you out of your mind. if you're thinking you drink because you're happy, you can't do it, whatever |  |
|  | Cognitive defusion | If they are suffering from depression or something, sometimes they use alcohol just to like, get away from their purpose. But it's not because the next day is still the problem, is there? It cannot help anymore. |  |
|  | Acceptance | The program helps me. You can track your stress and everything, in my mind, it was good. It's like a rehab. I like the one where I go to nature and just think and observe, and the switch can ease my mind, I can relax and some of the stress. |  |
|  | Acceptance | Ah, yes. One of them is about the for the exercise for the breathing exercise, I like that one. It makes me calm like this. How the exercise is being done so of course, as a domestic helper, stress is coming like that, so it helps. That's not the kind of exercise that comes to me down stress, but some of it also. It's hard. |  |
|  | Acceptance | It was great, it helps me with so many things as sessions like the one observing nature and going to places, and just thinking and observing |  |
| **Value-driven action** | Value clarification | Oh, it benefits me that life should be controlled. I should control my thinking…I should manage my emotions too much. |  |
|  | Value clarification | I need to become healthy because, like me, I am working, so I need to be healthy physically, mentally, and emotionally |  |
|  | Value clarification | I think drinking alcohol like that. Or learn how to control yourself or avoid drinking alcohol for our healthy body. Yeah. Yes, I know. This is appropriate |  |
|  | Purpose alignment | I will encourage others to read it carefully to help them. So, yeah, if they are suffering like depression or something, because sometimes they usually use alcohol to like just to like, get away from their purpose. But it's not because the next day is still the problem, is there. It cannot help anymore. |  |
|  | Goal setting | Yeah, it's useful because it helped me to confess to reach my goal, that is, not drinking alcohol. |  |
