# Supplementary material for: Effect of Integrated Internet-Based Acceptance and Commitment Therapy and Behavioral Activation Among Ethnic Minority Young Adults With Alcohol Use Disorder in Hong Kong: Pilot Randomized Controlled Trial
Source: J Med Internet Res. 2026 May 21;28:e83896. doi: 10.2196/83896 (PMC13193706; doi:10.2196/83896)
Supplement: Checklist 1 [file jmir-v28-e83896-s002.pdf]

CONSORT-EHEALTH (V 1.6.1) - Submission/Publication Form

The CONSORT-EHEALTH checklist is intended for authors of randomized trials evaluating web-based and Internet-based applications/interventions, including mobile interventions, electronic games (incl multiplayer games), social media, certain telehealth applications, and other interactive and/or networked electronic applications. Some of the items (e.g. all subitems under item 5 - description of the intervention) may also be applicable for other study designs.

The goal of the CONSORT EHEALTH checklist and guideline is to be
a) a guide for reporting for authors of RCTs,
b) to form a basis for appraisal of an ehealth trial (in terms of validity)

CONSORT-EHEALTH items/subitems are MANDATORY reporting items for studies published in the Journal of Medical Internet Research and other journals / scientific societies endorsing the checklist.

Items numbered 1., 2., 3., 4a., 4b etc are original CONSORT or CONSORT-NPT (non-pharmacologic treatment) items.
Items with Roman numerals (i., ii, iii, iv etc.) are CONSORT-EHEALTH extensions/clarifications.

As the CONSORT-EHEALTH checklist is still considered in a formative stage, we would ask that you also RATE ON A SCALE OF 1-5 how important/useful you feel each item is FOR THE PURPOSE OF THE CHECKLIST and reporting guideline (optional).

Mandatory reporting items are marked with a red *.
In the textboxes, either copy & paste the relevant sections from your manuscript into this form - please include any quotes from your manuscript in QUOTATION MARKS,
or answer directly by providing additional information not in the manuscript, or elaborating on why the item was not relevant for this study.

YOUR ANSWERS WILL BE PUBLISHED AS A SUPPLEMENTARY FILE TO YOUR PUBLICATION IN JMIR AND ARE CONSIDERED PART OF YOUR PUBLICATION (IF ACCEPTED).
Please fill in these questions diligently. Information will not be copyedited, so please use proper spelling and grammar, use correct capitalization, and avoid abbreviations.

DO NOT FORGET TO SAVE AS PDF _AND_ CLICK THE SUBMIT BUTTON SO YOUR ANSWERS ARE IN OUR DATABASE !!!

Your response is too large. Try shortening some answers.

Eysenbach G, CONSORT-EHEALTH Group

CONSORT-EHEALTH: Improving and Standardizing Evaluation Reports of Web-based and Mobile Health Interventions
J Med Internet Res 2011;13(4):e126
URL: http://www.jmir.org/2011/4/e126/
doi: 10.2196/jmir.1923
PMID: 22209829

getanehmulua@gmail.com Switch account

Not shared

* Indicates required question

Your name *

First Last

Ka Yan Ho

Primary Affiliation (short), City, Country *

University of Toronto, Toronto, Canada

Hong Kong Polytechnic University, Hong Kong

Your e-mail address *

abc@gmail.com

Kyeva.ho@polyu.edu.hk

Your response is too large. Try shortening some answers.

Title of your manuscript *

Provide the (draft) title of your manuscript.

Effect of Integrated Internet-Based Acceptance and Commitment Therapy and Behavioral Activation Among Ethnic Minority Young Adults with Alcohol Use Disorder in Hong Kong: A Pilot Randomized Controlled Trial

Name of your App/Software/Intervention *

If there is a short and a long/alternate name, write the short name first and add the long name in brackets.

ACT-BA intervention

Evaluated Version (if any)

e.g. "V1", "Release 2017-03-01", "Version 2.0.27913"

Your answer

Language(s) *

What language is the intervention/app in? If multiple languages are available, separate by comma (e.g. "English, French")

English

URL of your Intervention Website or App

e.g. a direct link to the mobile app on app in appstore (itunes, Google Play), or URL of the website. If the intervention is a DVD or hardware, you can also link to an Amazon page.

Your answer

Your response is too large. Try shortening some answers.

URL of an image/screenshot (optional)

Your answer

Accessibility *

Can an enduser access the intervention presently?

- (selected) access is free and open
- access only for special usergroups, not open
- access is open to everyone, but requires payment/subscription/in-app purchases
- app/intervention no longer accessible
- Other:

Primary Medical Indication/Disease/Condition *

e.g. "Stress", "Diabetes", or define the target group in brackets after the condition, e.g. "Autism (Parents of children with)", "Alzheimers (Informal Caregivers of)"

Alcohol use disorder(Ethnc minority young adu

Primary Outcomes measured in trial *

comma-separated list of primary outcomes reported in the trial

Feseability Measures

Your response is too large. Try shortening some answers.

Secondary/other outcomes

Are there any other outcomes the intervention is expected to affect?

Effectiveness on alcohol use-related outcomes

Recommended "Dose" *

What do the instructions for users say on how often the app should be used?

- ○ Approximately Daily
- ● Approximately Weekly
- ○ Approximately Monthly
- ○ Approximately Yearly
- ○ "as needed"
- ○ Other:

Your response is too large. Try shortening some answers.

Approx. Percentage of Users (starters) still using the app as recommended after 3 months *

- [x] unknown / not evaluated
- [ ] 0-10%
- [ ] 11-20%
- [ ] 21-30%
- [ ] 31-40%
- [ ] 41-50%
- [ ] 51-60%
- [ ] 61-70%
- [ ] 71%-80%
- [ ] 81-90%
- [ ] 91-100%
- [ ] Other:

Overall, was the app/intervention effective? *

- [ ] yes: all primary outcomes were significantly better in intervention group vs control
- [x] partly: SOME primary outcomes were significantly better in intervention group vs control
- [ ] no statistically significant difference between control and intervention
- [ ] potentially harmful: control was significantly better than intervention in one or more outcomes
- [ ] inconclusive: more research is needed

Your response is too large. Try shortening some answers.

Article Preparation Status/Stage *

At which stage in your article preparation are you currently (at the time you fill in this form)

- ( ) not submitted yet - in early draft status
- ( ) not submitted yet - in late draft status, just before submission
- ( ) submitted to a journal but not reviewed yet
- (•) submitted to a journal and after receiving initial reviewer comments
- ( ) submitted to a journal and accepted, but not published yet
- ( ) published
- ( ) Other:

Journal *

If you already know where you will submit this paper (or if it is already submitted), please provide the journal name (if it is not JMIR, provide the journal name under "other")

- ( ) not submitted yet / unclear where I will submit this
- (•) Journal of Medical Internet Research (JMIR)
- ( ) JMIR mHealth and UHealth
- ( ) JMIR Serious Games
- ( ) JMIR Mental Health
- ( ) JMIR Public Health
- ( ) JMIR Formative Research
- ( ) Other JMIR sister journal
- ( ) Other:

Your response is too large. Try shortening some answers.

Is this a full powered effectiveness trial or a pilot/feasibility trial? *

- (selected) Pilot/feasibility
- Fully powered

Manuscript tracking number *

If this is a JMIR submission, please provide the manuscript tracking number under "other" (The ms tracking number can be found in the submission acknowledgement email, or when you login as author in JMIR. If the paper is already published in JMIR, then the ms tracking number is the four-digit number at the end of the DOI, to be found at the bottom of each published article in JMIR)

- no ms number (yet) / not (yet) submitted to / published in JMIR
- (selected) Other: 83896

TITLE AND ABSTRACT

1a) TITLE: Identification as a randomized trial in the title

1a) Does your paper address CONSORT item 1a? *

I.e does the title contain the phrase "Randomized Controlled Trial"? (if not, explain the reason under "other")

- (selected) yes
- Other:

Your response is too large. Try shortening some answers.

1a-i) Identify the mode of delivery in the title

Identify the mode of delivery. Preferably use “web-based” and/or “mobile” and/or “electronic game” in the title. Avoid ambiguous terms like “online”, “virtual”, “interactive”. Use “Internet-based” only if Intervention includes non-web-based Internet components (e.g. email), use “computer-based” or “electronic” only if offline products are used. Use “virtual” only in the context of “virtual reality” (3-D worlds). Use “online” only in the context of “online support groups”. Complement or substitute product names with broader terms for the class of products (such as “mobile” or “smart phone” instead of “iphone”), especially if the application runs on different platforms.

	1	2	3	4	5	
subitem not at all important	○	○	○	○	◉	essential

Clear selection

Does your paper address subitem 1a-i? *

Copy and paste relevant sections from manuscript title (include quotes in quotation marks "like this" to indicate direct quotes from your manuscript), or elaborate on this item by providing additional information not in the ms, or briefly explain why the item is not applicable/relevant for your study

"Integrated Internet-Based Acceptance and Commitment
Therapy and Behavioral Activation "

1a-ii) Non-web-based components or important co-interventions in title

Mention non-web-based components or important co-interventions in title, if any (e.g., “with telephone support”).

	1	2	3	4	5	
subitem not at all important	◉	○	○	○	○	essential

Clear selection

Your response is too large. Try shortening some answers.

Does your paper address subitem 1a-ii?

Copy and paste relevant sections from manuscript title (include quotes in quotation marks "like this" to indicate direct quotes from your manuscript), or elaborate on this item by providing additional information not in the ms, or briefly explain why the item is not applicable/relevant for your study

Your answer

1a-iii) Primary condition or target group in the title

Mention primary condition or target group in the title, if any (e.g., “for children with Type I Diabetes”) Example: A Web-based and Mobile Intervention with Telephone Support for Children with Type I Diabetes: Randomized Controlled Trial

	1	2	3	4	5	
subitem not at all important	○	◉	○	○	○	essential

Clear selection

Does your paper address subitem 1a-iii? *

Copy and paste relevant sections from manuscript title (include quotes in quotation marks "like this" to indicate direct quotes from your manuscript), or elaborate on this item by providing additional information not in the ms, or briefly explain why the item is not applicable/relevant for your study

"Ethnic Minority Young Adults with Alcohol Use Disorder"

1b) ABSTRACT: Structured summary of trial design, methods, results, and conclusions

NPT extension: Description of experimental treatment, comparator, care providers, centers, and blinding status.

Your response is too large. Try shortening some answers.

1b-i) Key features/functionalities/components of the intervention and comparator in the METHODS section of the ABSTRACT

Mention key features/functionalities/components of the intervention and comparator in the abstract. If possible, also mention theories and principles used for designing the site. Keep in mind the needs of systematic reviewers and indexers by including important synonyms. (Note: Only report in the abstract what the main paper is reporting. If this information is missing from the main body of text, consider adding it)

	1	2	3	4	5	
subitem not at all important	○	○	○	○	◉	essential

Clear selection

Does your paper address subitem 1b-i? *

Copy and paste relevant sections from the manuscript abstract (include quotes in quotation marks "like this" to indicate direct quotes from your manuscript), or elaborate on this item by providing additional information not in the ms, or briefly explain why the item is not applicable/relevant for your study

"40 participants were randomly assigned to either the ACT-BA or Treatment-as-Usual (TAU) group."

1b-ii) Level of human involvement in the METHODS section of the ABSTRACT

Clarify the level of human involvement in the abstract, e.g., use phrases like “fully automated” vs. “therapist/nurse/care provider/physician-assisted” (mention number and expertise of providers involved, if any). (Note: Only report in the abstract what the main paper is reporting. If this information is missing from the main body of text, consider adding it)

	1	2	3	4	5	
subitem not at all important	○	○	○	○	◉	essential

Your response is too large. Try shortening some answers.

Does your paper address subitem 1b-ii?

Copy and paste relevant sections from the manuscript abstract (include quotes in quotation marks "like this" to indicate direct quotes from your manuscript), or elaborate on this item by providing additional information not in the ms, or briefly explain why the item is not applicable/relevant for your study

Your answer

1b-iii) Open vs. closed, web-based (self-assessment) vs. face-to-face assessments in the METHODS section of the ABSTRACT

Mention how participants were recruited (online vs. offline), e.g., from an open access website or from a clinic or a closed online user group (closed usergroup trial), and clarify if this was a purely web-based trial, or there were face-to-face components (as part of the intervention or for assessment). Clearly say if outcomes were self-assessed through questionnaires (as common in web-based trials). Note: In traditional offline trials, an open trial (open-label trial) is a type of clinical trial in which both the researchers and participants know which treatment is being administered. To avoid confusion, use “blinded” or “unblinded” to indicated the level of blinding instead of “open”, as “open” in web-based trials usually refers to “open access” (i.e. participants can self-enrol). (Note: Only report in the abstract what the main paper is reporting. If this information is missing from the main body of text, consider adding it)

	1	2	3	4	5	
subitem not at all important	○	○	○	○	◉	essential

Clear selection

Does your paper address subitem 1b-iii?

Copy and paste relevant sections from the manuscript abstract (include quotes in quotation marks "like this" to indicate direct quotes from your manuscript), or elaborate on this item by providing additional information not in the ms, or briefly explain why the item is not applicable/relevant for your study

Your answer

Your response is too large. Try shortening some answers.

1b-iv) RESULTS section in abstract must contain use data

Report number of participants enrolled/assessed in each group, the use/uptake of the intervention (e.g., attrition/adherence metrics, use over time, number of logins etc.), in addition to primary/secondary outcomes. (Note: Only report in the abstract what the main paper is reporting. If this information is missing from the main body of text, consider adding it)

	1	2	3	4	5	
subitem not at all important	○	○	○	○	●	essential

Clear selection

Does your paper address subitem 1b-iv?

Copy and paste relevant sections from the manuscript abstract (include quotes in quotation marks "like this" to indicate direct quotes from your manuscript), or elaborate on this item by providing additional information not in the ms, or briefly explain why the item is not applicable/relevant for your study

Your answer

1b-v) CONCLUSIONS/DISCUSSION in abstract for negative trials

Conclusions/Discussions in abstract for negative trials: Discuss the primary outcome - if the trial is negative (primary outcome not changed), and the intervention was not used, discuss whether negative results are attributable to lack of uptake and discuss reasons. (Note: Only report in the abstract what the main paper is reporting. If this information is missing from the main body of text, consider adding it)

	1	2	3	4	5	
subitem not at all important	○	○	○	○	●	essential

Clear selection

Your response is too large. Try shortening some answers.

Does your paper address subitem 1b-v?

Copy and paste relevant sections from the manuscript abstract (include quotes in quotation marks "like this" to indicate direct quotes from your manuscript), or elaborate on this item by providing additional information not in the ms, or briefly explain why the item is not applicable/relevant for your study

Your answer

INTRODUCTION

2a) In INTRODUCTION: Scientific background and explanation of rationale

2a-i) Problem and the type of system/solution

Describe the problem and the type of system/solution that is object of the study: intended as stand-alone intervention vs. incorporated in broader health care program? Intended for a particular patient population? Goals of the intervention, e.g., being more cost-effective to other interventions, replace or complement other solutions? (Note: Details about the intervention are provided in "Methods" under 5)

	1	2	3	4	5	
subitem not at all important	○	○	○	○	◉	essential

Clear selection

Your response is too large. Try shortening some answers.

Does your paper address subitem 2a-i? *

Copy and paste relevant sections from the manuscript (include quotes in quotation marks "like this" to indicate direct quotes from your manuscript), or elaborate on this item by providing additional information not in the ms, or briefly explain why the item is not applicable/relevant for your study

Your response is too large. Try shortening some answers.

"Acceptance and Commitment Therapy (ACT) has emerged as a promising intervention for substance use and mental health disorders, enhancing psychological flexibility (45, 48). Grounded in Relational Frame Theory (RFT) (49-51), ACT enhances conscious behavioral adaptation aligned with personal values while accepting negative thoughts and emotions (52). Unlike traditional therapies, ACT focuses on behavioral change over symptom reduction, making it particularly suitable for avoidance-driven conditions like AUD (49, 51, 53). Literature review suggests that ACT may be a viable treatment option for ethnic minority young adults with AUD (47, 54-56).
However, our review identified methodological limitations in existing studies on the effectiveness of ACT on AUD and substantial challenges in engagement and adherence to ACT (57-59). Firstly, many studies lacked appropriate control groups and failed to directly assess alcohol-related outcomes (e.g., drinking days, drinks per drinking day, heavy drinking days, and abstinence duration). Second, for the adherence issues, participants showed low engagement with value-based activities for guiding committed action toward a meaningful life (57, 58). This is potentially attributed to difficulty in identifying enjoyable, rewarding alcohol-free activities and overemphasis on internal experiences (emotions/thoughts) without sufficient attention to overt behaviors. Consequently, these limitations warrant integrating novel strategies into ACT to enhance engagement and adherence.
Behavioral activation (BA) may be a possible method to overcome engagement and adherence problems in ACT. BA is a widely recognized and theoretically sound approach that has been applied to treat depression (62, 63). Its application has been extended to substance use disorders (SUD) via providing rewarding experiences in daily life.
from substance use, thus achieving abstinence and preventing relapse (64-66). Evidence suggests that BA is a promising intervention for SUD as a standalone treatment or when integrated with other therapeutic modalities. The emphasis on rewarding experiences in BA exactly addresses the low engagement and adherence of ACT due to the failure to identify enjoyable, rewarding, and positively reinforcing activities in the intervention process. Because of the distinctive benefits of BA, it appears to be a treatment component complementary to the shortcomings of ACT, thus enhancing the intervention effectiveness if They both are integrated into the treatment protocol. Also, behavioral activation is a brief intervention that can be delivered within 4 weeks, which will not significantly prolong the duration of each intervention session, ensuring the participants' adherence and engagement. In sum, ACT simply encourages an individual to ACT, which includes Accepting the reactions and being present, Choosing a valued direction, and taking actions while BA motivates an individual to take real actions. Two studies have integrated BA into ACT to manage different conditions in various populations, including an internet-based behavioral activation and acceptance-based treatment for depression (67) and an open trial of a new acceptance-based behavioral treatment for major depression and psychotic features (65). The results were promising, which supported that the integration is not only theoretically sound but also feasible and, importantly, can translate into observable benefits.
Regarding mode of intervention delivery, our qualitative finding revealed significant treatment barriers among ethnic minority young adults, in which no participant had accessed treatments for AUD due to their engagement in work. Apart from work, their time and financial constraints also hindered them from seeking hospital-based care (26).
Internet-based interventions may be a viable option to address these challenges by

Your response is too large. Try shortening some answers.

[illegible]
supports this modality (73, 75). However, no studies have evaluated internet-based self-help
[illegible]

interventions integrating ACT and BA for these populations Therefore this study examined
interventions integrating ACT and BA for these populations. Therefore, this study examined the preliminary effectiveness and feasibility of internet-based self-help integrated ACT and BA intervention in improving alcohol use abstinence among ethnic minority young adults with AUD."

2a-ii) Scientific background, rationale: What is known about the (type of) system

Scientific background, rationale: What is known about the (type of) system that is the object of the study (be sure to discuss the use of similar systems for other conditions/diagnoses, if appropiate), motivation for the study, i.e. what are the reasons for and what is the context for this specific study, from which stakeholder viewpoint is the study performed, potential impact of findings [2]. Briefly justify the choice of the comparator.

	1	2	3	4	5	
subitem not at all important	○	○	○	○	◉	essential

Clear selection

Your response is too large. Try shortening some answers.

Does your paper address subitem 2a-ii? *

Copy and paste relevant sections from the manuscript (include quotes in quotation marks "like this" to indicate direct quotes from your manuscript), or elaborate on this item by providing additional information not in the ms, or briefly explain why the item is not applicable/relevant for your study

Your response is too large. Try shortening some answers.

"AUD remains a significant public health issue, disproportionately affecting ethnic minority young adults compared to the general population (16-18). In Hong Kong, ethnic minorities constitute about 8.4% of the population (19, 20). However, unlike locals, they encounter various challenges, including stigma, discrimination, acculturation stress, low self-esteem, social integration difficulties, and loneliness, contributing to unhealthy drinking behaviors and AUD (7, 21-27). Previous studies showed that over 50% of ethnic minorities have less access to education and employment compared to the Chinese population, and about 60% of Chinese respondents believed ethnic minorities should be limited to low-skilled occupations (28). Our previous empirical evidence further identifies cultural factors, family influences, hedonistic motives, curiosity, low-risk perception, coping motives, social pressures, and subjective cravings as reasons behind AUD development among ethnic minority young adults (29). Acceptance and Commitment Therapy (ACT) has emerged as a promising intervention for substance use and mental health disorders, enhancing psychological flexibility (45, 48). Grounded in Relational Frame Theory (RFT) (49-51), ACT enhances conscious behavioral adaptation aligned with personal values while accepting negative thoughts and emotions (52). Unlike traditional therapies, ACT focuses on behavioral change over symptom reduction, making it particularly suitable for avoidance-driven conditions like AUD (49, 51, 53). Literature review suggests that ACT may be a viable treatment option for ethnic minority young adults with AUD (47, 54-56). However, our review identified methodological limitations in existing studies on the effectiveness of ACT on AUD and substantial challenges in engagement and adherence to ACT (57-59). Firstly, many studies lacked appropriate control groups and failed to directly assess alcohol-related outcomes (e.g., drinking days, drinks per drinking day, heavy drinking days, and abstinence duration). Second, for the adherence issues, participants showed low engagement with value-based activities for guiding committed action toward a meaningful life (57, 58). This is potentially attributed to difficulty in identifying enjoyable, rewarding alcohol-free activities and overemphasis on internal experiences (emotions/thoughts) without sufficient attention to overt behaviors. Lastly, ACT does not contain any daily activity for practice, which is a critical challenge for ethnic minorities with AUD to identify pleasurable alternatives to replace drinking, leading to relapse despite quitting attempts. Consequently, these limitations warrant integrating novel strategies into ACT to enhance engagement and adherence."

Your response is too large. Try shortening some answers.

Does your paper address CONSORT subitem 2b? *

Copy and paste relevant sections from the manuscript (include quotes in quotation marks "like this" to indicate direct quotes from your manuscript), or elaborate on this item by providing additional information not in the ms, or briefly explain why the item is not applicable/relevant for your study

"This study examined the preliminary effectiveness and feasibility of an internet-based self-help program integrating ACT and BA on alcohol abstinence among Hong Kong ethnic minority young adults with AUD. The primary objective of this study was to evaluate the intervention feasibility in terms of various feasibility outcomes under CONSORT 2010, including rates of eligibility, consent, randomization, adherence, retention, completion, missing data, and adverse events.
The secondary objective was (1) to evaluate the preliminary effectiveness of this internet-based self-help program integrating ACT and BA on several outcomes, including drinking days, drinks per drinking day, heavy drinking days, cumulative abstinence duration, alcohol abstinence self-efficacy, psychological flexibility, readiness to change, and everyday discrimination in this population; and (2) to estimate the effect size of the ACT-BA intervention on these same outcomes."

METHODS

3a) Description of trial design (such as parallel, factorial) including allocation ratio

Your response is too large. Try shortening some answers.

Does your paper address CONSORT subitem 3a? *

Copy and paste relevant sections from the manuscript (include quotes in quotation marks "like this" to indicate direct quotes from your manuscript), or elaborate on this item by providing additional information not in the ms, or briefly explain why the item is not applicable/relevant for your study

"A single-blinded randomized controlled trial study was conducted among Hong Kong ethnic minority young adults with AUD. Stratified block randomization was implemented by a trained student assistant (SA) to allocate participants into intervention and control groups (20 per group). To ensure baseline balance of prognostic factors, particularly ethnicity, sex, and disease severity, which could confound the intervention effects if unevenly distributed, Participants were first stratified by these factors. Within each stratum, block randomization (1:1 ratio, block size of 4) was performed using a computer-based random number generator. Allocation sequences were concealed using a computer-generated random number. Due to the nature of the intervention and self-administered questionnaires, blinding of participants was not feasible. However, the outcome assessor was blinded to the participants' group assignments throughout the study."

3b) Important changes to methods after trial commencement (such as eligibility criteria), with reasons

Does your paper address CONSORT subitem 3b? *

Copy and paste relevant sections from the manuscript (include quotes in quotation marks "like this" to indicate direct quotes from your manuscript), or elaborate on this item by providing additional information not in the ms, or briefly explain why the item is not applicable/relevant for your study

"We did not change eligibility criteria after trial commencement."

Your response is too large. Try shortening some answers.

3b-i) Bug fixes, Downtimes, Content Changes

Bug fixes, Downtimes, Content Changes: ehealth systems are often dynamic systems. A description of changes to methods therefore also includes important changes made on the intervention or comparator during the trial (e.g., major bug fixes or changes in the functionality or content) (5-iii) and other “unexpected events” that may have influenced study design such as staff changes, system failures/downtimes, etc. [2].

	1	2	3	4	5	
subitem not at all important	○	○	○	○	◉	essential

Clear selection

Does your paper address subitem 3b-i?

Copy and paste relevant sections from the manuscript (include quotes in quotation marks "like this" to indicate direct quotes from your manuscript), or elaborate on this item by providing additional information not in the ms, or briefly explain why the item is not applicable/relevant for your study

Your answer

4a) Eligibility criteria for participants

Your response is too large. Try shortening some answers.

Does your paper address CONSORT subitem 4a? *

Copy and paste relevant sections from the manuscript (include quotes in quotation marks "like this" to indicate direct quotes from your manuscript), or elaborate on this item by providing additional information not in the ms, or briefly explain why the item is not applicable/relevant for your study

"Participants who met the following criteria were considered for this study: (1) Young adults (18 to 35 years) from ethnic minorities in Hong Kong; (2) proficient in English (read, write, and speak English); (3) voluntary participants; (4) possessing internet-enabled smartphones or electronic devices; and (5) meeting the Diagnostic and Statistical Manual of Mental Disorders, Fifth Edition (DSM-5) criteria for AUD, defined as exhibiting at least two of the 11 symptoms specified in the DSM-5 (84). Ethnic minorities in this age range were chosen, as it aligns with the US Census Bureau classification for young adults (85), and this age group is the population group with the highest vulnerability for substance use disorders (86)."

4a-i) Computer / Internet literacy

Computer / Internet literacy is often an implicit “de facto” eligibility criterion - this should be explicitly clarified.

	1	2	3	4	5	
subitem not at all important	○	○	○	○	◉	essential

Clear selection

Does your paper address subitem 4a-i?

Copy and paste relevant sections from the manuscript (include quotes in quotation marks "like this" to indicate direct quotes from your manuscript), or elaborate on this item by providing additional information not in the ms, or briefly explain why the item is not applicable/relevant for your study

Your response is too large. Try shortening some answers.

4a-ii) Open vs. closed, web-based vs. face-to-face assessments:

Open vs. closed, web-based vs. face-to-face assessments: Mention how participants were recruited (online vs. offline), e.g., from an open access website or from a clinic, and clarify if this was a purely web-based trial, or there were face-to-face components (as part of the intervention or for assessment), i.e., to what degree got the study team to know the participant. In online-only trials, clarify if participants were quasi-anonymous and whether having multiple identities was possible or whether technical or logistical measures (e.g., cookies, email confirmation, phone calls) were used to detect/prevent these.

	1	2	3	4	5	
subitem not at all important	○	○	○	○	◉	essential

Clear selection

Does your paper address subitem 4a-ii? *

Copy and paste relevant sections from the manuscript (include quotes in quotation marks "like this" to indicate direct quotes from your manuscript), or elaborate on this item by providing additional information not in the ms, or briefly explain why the item is not applicable/relevant for your study

"Participants were recruited in the community through face-to-face approaches at bars, communal recreational areas, schools, and gardens where ethnic minorities usually gather."

4a-iii) Information giving during recruitment

Information given during recruitment. Specify how participants were briefed for recruitment and in the informed consent procedures (e.g., publish the informed consent documentation as appendix, see also item X26), as this information may have an effect on user self-selection, user expectation and may also bias results.

	1	2	3	4	5	
subitem not at all important	○	○	○	○	◉	essential

Clear selection

Your response is too large. Try shortening some answers.

Does your paper address subitem 4a-iii?

Copy and paste relevant sections from the manuscript (include quotes in quotation marks "like this" to indicate direct quotes from your manuscript), or elaborate on this item by providing additional information not in the ms, or briefly explain why the item is not applicable/relevant for your study

"The RA approached the potential ethnic minorities, introduced the study, and gave them a poster to read the details, objectives, and eligibility criteria of the study. Interested ethnic minorities were instructed to scan the QR code and complete a screening in a Google form to determine their eligibility. A brief session was conducted to guide the participants to access the intervention using their smart devices during subject recruitment."

4b) Settings and locations where the data were collected

Does your paper address CONSORT subitem 4b? *

Copy and paste relevant sections from the manuscript (include quotes in quotation marks "like this" to indicate direct quotes from your manuscript), or elaborate on this item by providing additional information not in the ms, or briefly explain why the item is not applicable/relevant for your study

"This study was conducted in Hong Kong, a special Chinese administrative region located east of the Pearl River Delta. Participants were recruited in the community through face-to-face approaches at bars, communal recreational areas, schools, and gardens where ethnic minorities usually gather."

Your response is too large. Try shortening some answers.

4b-i) Report if outcomes were (self-)assessed through online questionnaires

Clearly report if outcomes were (self-)assessed through online questionnaires (as common in web-based trials) or otherwise.

	1	2	3	4	5	
subitem not at all important	○	○	○	○	●	essential

Clear selection

Does your paper address subitem 4b-i? *

Copy and paste relevant sections from the manuscript (include quotes in quotation marks "like this" to indicate direct quotes from your manuscript), or elaborate on this item by providing additional information not in the ms, or briefly explain why the item is not applicable/relevant for your study

"Participants were then provided a personal link to access the Qualtrics website. They were provided instructions to complete baseline data. Consequently, participants were randomly assigned to either the ACT-BA group or the TAU group. Quantitative data were collected at two time points, i.e., baseline and post-intervention online."

4b-ii) Report how institutional affiliations are displayed

Report how institutional affiliations are displayed to potential participants [on ehealth media], as affiliations with prestigious hospitals or universities may affect volunteer rates, use, and reactions with regards to an intervention.(Not a required item – describe only if this may bias results)

	1	2	3	4	5	
subitem not at all important	○	○	○	○	●	essential

Clear selection

Your response is too large. Try shortening some answers.

Does your paper address subitem 4b-ii?

Copy and paste relevant sections from the manuscript (include quotes in quotation marks "like this" to indicate direct quotes from your manuscript), or elaborate on this item by providing additional information not in the ms, or briefly explain why the item is not applicable/relevant for your study

Your answer

5) The interventions for each group with sufficient details to allow replication, including how and when they were actually administered

5-i) Mention names, credential, affiliations of the developers, sponsors, and owners

Mention names, credential, affiliations of the developers, sponsors, and owners [6] (if authors/evaluators are owners or developer of the software, this needs to be declared in a "Conflict of interest" section or mentioned elsewhere in the manuscript).

	1	2	3	4	5	
subitem not at all important	○	○	○	○	○	essential

Does your paper address subitem 5-i?

Copy and paste relevant sections from the manuscript (include quotes in quotation marks "like this" to indicate direct quotes from your manuscript), or elaborate on this item by providing additional information not in the ms, or briefly explain why the item is not applicable/relevant for your study

Your answer

Your response is too large. Try shortening some answers.

5-ii) Describe the history/development process

Describe the history/development process of the application and previous formative evaluations (e.g., focus groups, usability testing), as these will have an impact on adoption/use rates and help with interpreting results.

	1	2	3	4	5	
subitem not at all important	○	○	○	○	○	essential

Does your paper address subitem 5-ii?

Copy and paste relevant sections from the manuscript (include quotes in quotation marks "like this" to indicate direct quotes from your manuscript), or elaborate on this item by providing additional information not in the ms, or briefly explain why the item is not applicable/relevant for your study

Your answer

5-iii) Revisions and updating

Revisions and updating. Clearly mention the date and/or version number of the application/intervention (and comparator, if applicable) evaluated, or describe whether the intervention underwent major changes during the evaluation process, or whether the development and/or content was “frozen” during the trial. Describe dynamic components such as news feeds or changing content which may have an impact on the replicability of the intervention (for unexpected events see item 3b).

	1	2	3	4	5	
subitem not at all important	○	○	○	○	○	essential

Your response is too large. Try shortening some answers.

Does your paper address subitem 5-iii?

Copy and paste relevant sections from the manuscript (include quotes in quotation marks "like this" to indicate direct quotes from your manuscript), or elaborate on this item by providing additional information not in the ms, or briefly explain why the item is not applicable/relevant for your study

Your answer

5-iv) Quality assurance methods

Provide information on quality assurance methods to ensure accuracy and quality of information provided [1], if applicable.

	1	2	3	4	5	
subitem not at all important	○	○	○	○	○	essential

Does your paper address subitem 5-iv?

Copy and paste relevant sections from the manuscript (include quotes in quotation marks "like this" to indicate direct quotes from your manuscript), or elaborate on this item by providing additional information not in the ms, or briefly explain why the item is not applicable/relevant for your study

Your answer

Your response is too large. Try shortening some answers.

5-v) Ensure replicability by publishing the source code, and/or providing screenshots/screen-capture video, and/or providing flowcharts of the algorithms used

Ensure replicability by publishing the source code, and/or providing screenshots/screen-capture video, and/or providing flowcharts of the algorithms used. Replicability (i.e., other researchers should in principle be able to replicate the study) is a hallmark of scientific reporting.

	1	2	3	4	5	
subitem not at all important	○	○	○	○	○	essential

Does your paper address subitem 5-v?

Copy and paste relevant sections from the manuscript (include quotes in quotation marks "like this" to indicate direct quotes from your manuscript), or elaborate on this item by providing additional information not in the ms, or briefly explain why the item is not applicable/relevant for your study

Your answer

5-vi) Digital preservation

Digital preservation: Provide the URL of the application, but as the intervention is likely to change or disappear over the course of the years; also make sure the intervention is archived (Internet Archive, webcitation.org, and/or publishing the source code or screenshots/videos alongside the article). As pages behind login screens cannot be archived, consider creating demo pages which are accessible without login.

	1	2	3	4	5	
subitem not at all important	○	○	○	○	○	essential

Your response is too large. Try shortening some answers.

Does your paper address subitem 5-vi?

Copy and paste relevant sections from the manuscript (include quotes in quotation marks "like this" to indicate direct quotes from your manuscript), or elaborate on this item by providing additional information not in the ms, or briefly explain why the item is not applicable/relevant for your study

Your answer

5-vii) Access

Access: Describe how participants accessed the application, in what setting/context, if they had to pay (or were paid) or not, whether they had to be a member of specific group. If known, describe how participants obtained "access to the platform and Internet" [1]. To ensure access for editors/reviewers/readers, consider to provide a "backdoor" login account or demo mode for reviewers/readers to explore the application (also important for archiving purposes, see vi).

	1	2	3	4	5	
subitem not at all important	○	○	○	○	◉	essential

Clear selection

Does your paper address subitem 5-vii? *

Copy and paste relevant sections from the manuscript (include quotes in quotation marks "like this" to indicate direct quotes from your manuscript), or elaborate on this item by providing additional information not in the ms, or briefly explain why the item is not applicable/relevant for your study

"Participants were then provided a personal link to access the Qualtrics website. They were provided instructions to complete baseline data. Consequently, participants were randomly assigned to either the ACT-BA group or the TAU group. Quantitative data were collected at two time points, i.e., baseline and post-intervention online."

Your response is too large. Try shortening some answers.

5-viii) Mode of delivery, features/functionalities/components of the intervention and comparator, and the theoretical framework

Describe mode of delivery, features/functionalities/components of the intervention and comparator, and the theoretical framework [6] used to design them (instructional strategy [1], behaviour change techniques, persuasive features, etc., see e.g., [7, 8] for terminology). This includes an in-depth description of the content (including where it is coming from and who developed it) [1]," whether [and how] it is tailored to individual circumstances and allows users to track their progress and receive feedback" [6]. This also includes a description of communication delivery channels and – if computer-mediated communication is a component – whether communication was synchronous or asynchronous [6]. It also includes information on presentation strategies [1], including page design principles, average amount of text on pages, presence of hyperlinks to other resources, etc. [1].

	1	2	3	4	5	
subitem not at all important	○	○	○	○	◉	essential

Clear selection

Your response is too large. Try shortening some answers.

Does your paper address subitem 5-viii? *

Copy and paste relevant sections from the manuscript (include quotes in quotation marks "like this" to indicate direct quotes from your manuscript), or elaborate on this item by providing additional information not in the ms, or briefly explain why the item is not applicable/relevant for your study

Your response is too large. Try shortening some answers.

"The intervention contained a total of six weekly individual sessions structured sequentially. A research assistant in the team sent text reminders to the participants via different information communication systems, e.g., WhatsApp and WeChat one day before the intervention session, ensure their attendance. For session 1, it aimed to establish the therapeutic relationship with the participants and assess the participants' experiential avoidance behaviors. In this session, participants learned through multimedia resources what avoidance behavior was and how it impacted their life in terms of different perspectives,
including family, health, work, social life, and the community and withdrawal symptoms. They also identified alcohol-free rewarding activities, and initiated daily monitoring through rating enjoyment or importance (from 1 to 10) in a workbook. High-scoring activities were considered for further value-driven planning. Session 2 focused on value identification in which participants learned and identified core values across life domains and developed corresponding value-driven activity plans using a structured worksheet. They scheduled at at least one value-congruent activity per domain. Session 3 introduced the concept of acceptance and cognitive defusion. Participants learned and practiced various acceptance and diffusion skills and mindfulness exercises to manage experiential avoidance, which was usually managed by drinking. They learned different techniques of acceptance by (1) letting feelings, urges, and thoughts occur without a desire to act on them, (2) noticing their strengths while acknowledging their shortcomings, and (3) facing the difficulty without running away from or avoiding it. They also learned defusion skills by considering their thoughts as thoughts only that could come and go, and sometimes they're impulsive thoughts
about drinking were not facts. Session 4 focused on present moment awareness via mindful practices. Participants learned to observe internal experiences (such as cravings, negative emotions, and thoughts) related to past or future events that led to AUD. Observing self-skills was also developed through metaphors. Particularly, the participants learned how to consider themselves, and hence understood that there was a part of them who could think, feel, notice, and observe their cravings and feelings toward drinking. Session 5 reinforced value-directed behavioral activation, with participants identifying new value-congruent alcohol-free activities and integrating them into actionable daily plans and taking committed action towards their life goals. Session 6 emphasized the post-treatment plan and relapse prevention strategies. Previous sessions were reviewed, and another long-term goal congruent with their values was developed.
Control group
The control group, referred to as Treatment-as-Usual (TAU), received standard or usual community care without any active interventions from the study. In previous studies, such standards have been used as a benchmark for comparison with ACT (89-91). Participants continued the existing community services that they have already engaged in. Nevertheless, they were asked to report the received services to the research team during the study period."

Your response is too large. Try shortening some answers.

5-ix) Describe use parameters

Describe use parameters (e.g., intended “doses” and optimal timing for use). Clarify what instructions or recommendations were given to the user, e.g., regarding timing, frequency, heaviness of use, if any, or was the intervention used ad libitum.

	1	2	3	4	5	
subitem not at all important	○	○	○	○	○	essential

Does your paper address subitem 5-ix?

Copy and paste relevant sections from the manuscript (include quotes in quotation marks "like this" to indicate direct quotes from your manuscript), or elaborate on this item by providing additional information not in the ms, or briefly explain why the item is not applicable/relevant for your study

Your answer

5-x) Clarify the level of human involvement

Clarify the level of human involvement (care providers or health professionals, also technical assistance) in the e-intervention or as co-intervention (detail number and expertise of professionals involved, if any, as well as “type of assistance offered, the timing and frequency of the support, how it is initiated, and the medium by which the assistance is delivered”. It may be necessary to distinguish between the level of human involvement required for the trial, and the level of human involvement required for a routine application outside of a RCT setting (discuss under item 21 – generalizability).

	1	2	3	4	5	
subitem not at all important	○	○	○	○	○	essential

Your response is too large. Try shortening some answers.

Does your paper address subitem 5-x?

Copy and paste relevant sections from the manuscript (include quotes in quotation marks "like this" to indicate direct quotes from your manuscript), or elaborate on this item by providing additional information not in the ms, or briefly explain why the item is not applicable/relevant for your study

Your answer

5-xi) Report any prompts/reminders used

Report any prompts/reminders used: Clarify if there were prompts (letters, emails, phone calls, SMS) to use the application, what triggered them, frequency etc. It may be necessary to distinguish between the level of prompts/reminders required for the trial, and the level of prompts/reminders for a routine application outside of a RCT setting (discuss under item 21 – generalizability).

	1	2	3	4	5	
subitem not at all important	○	○	○	○	◉	essential

Clear selection

Does your paper address subitem 5-xi? *

Copy and paste relevant sections from the manuscript (include quotes in quotation marks "like this" to indicate direct quotes from your manuscript), or elaborate on this item by providing additional information not in the ms, or briefly explain why the item is not applicable/relevant for your study

"The intervention group received an internet-based self-help program that integrated both ACT and BA, using an online platform in Qualtrics, through a personalized link. The
The intervention contained a total of six weekly individual sessions structured sequentially. A
research assistant in the team sent text reminders to the participants via different information communication systems, e.g., WhatsApp and WeChat."

Your response is too large. Try shortening some answers.

5-xii) Describe any co-interventions (incl. training/support)

Describe any co-interventions (incl. training/support): Clearly state any interventions that are provided in addition to the targeted eHealth intervention, as ehealth intervention may not be designed as stand-alone intervention. This includes training sessions and support [1]. It may be necessary to distinguish between the level of training required for the trial, and the level of training for a routine application outside of a RCT setting (discuss under item 21 – generalizability.

	1	2	3	4	5	
subitem not at all important	○	○	○	○	◉	essential

Clear selection

Does your paper address subitem 5-xii? *

Copy and paste relevant sections from the manuscript (include quotes in quotation marks "like this" to indicate direct quotes from your manuscript), or elaborate on this item by providing additional information not in the ms, or briefly explain why the item is not applicable/relevant for your study

"Since the intervention modules are comprehensive, clear, simple, and self-help, additional Structured training or support was not provided.

6a) Completely defined pre-specified primary and secondary outcome measures, including how and when they were assessed

Your response is too large. Try shortening some answers.

Does your paper address CONSORT subitem 6a? *

Copy and paste relevant sections from the manuscript (include quotes in quotation marks "like this" to indicate direct quotes from your manuscript), or elaborate on this item by providing additional information not in the ms, or briefly explain why the item is not applicable/relevant for your study

Your response is too large. Try shortening some answers.

"Feasibility outcomes
The feasibility of this study was assessed by evaluating eligibility rate (number of eligible participants divided by number of screened ethnics minorities), consent rate (number of participants consented divided by number of eligible participants), randomization rate (number of participants who were randomized divided by number of participants who consented), adherence rate (number of participants in the intervention group who completed all intervention sessions divided by the number of participants randomised into the intervention group), retention rate (number of participants who remained in the study divided by number of participants who were randomized), completion rate (number of participants who answered the questionnaires divided by number of questionnaires being distributed), adverse events which were defined as unfavourable and unintended events that were absent from baseline or appeared to worsen from baseline during the study period, and missing data (the percentage of missing in the dataset).
Effectiveness outcomes
Drinking-related outcomes: The Timeline Follow-Back Questionnaire was used to assess the following drinking-related outcomes, including drinking days, drinks per drinking day, heavy drinking days, and cumulative abstinence duration. Every participant was asked to report the number of standard drinks (one unit contains 12 grams of pure alcohol) consumed within 30 days pre- and post-intervention. Then, the average number of drinks per drinking day, the number of drinking days and heavy drinking days, and cumulative abstinence were computed. A heavy drinking day is defined as having 4 or more drinks for females and 5 or more drinks on any day for males (92). The cumulative abstinence duration (CAD) was calculated by dividing the number of abstinent days by 30 days.
Alcohol abstinence self-efficacy: The self-efficacy of participants in alcohol abstinence was measured using the Alcohol Abstinence Self-Efficacy Scale (AASE).
Readiness to change: The participant's readiness to change in drinking was measured using the Readiness to Change Questionnaire (RTCQ), a 12-item instrument (93).
Psychological flexibility: The Acceptance and Action Questionnaire II (AAQ-II) was used to Measure psychological flexibility.
Everyday discrimination: The participants' everyday discrimination was measured using the short version of the Everyday Discrimination Scale postintervention (EDS) (95)."

Your response is too large. Try shortening some answers.

6a-i) Online questionnaires: describe if they were validated for online use and apply CHERRIES items to describe how the questionnaires were designed/deployed

If outcomes were obtained through online questionnaires, describe if they were validated for online use and apply CHERRIES items to describe how the questionnaires were designed/deployed [9].

	1	2	3	4	5	
subitem not at all important	○	○	○	○	○	essential

Does your paper address subitem 6a-i?

Copy and paste relevant sections from manuscript text

Your answer

6a-ii) Describe whether and how "use" (including intensity of use/dosage) was defined/measured/monitored

Describe whether and how "use" (including intensity of use/dosage) was defined/measured/monitored (logins, logfile analysis, etc.). Use/adoption metrics are important process outcomes that should be reported in any ehealth trial.

	1	2	3	4	5	
subitem not at all important	○	○	○	○	○	essential

Does your paper address subitem 6a-ii?

Copy and paste relevant sections from manuscript text

Your answer

Your response is too large. Try shortening some answers.

6a-iii) Describe whether, how, and when qualitative feedback from participants was obtained

Describe whether, how, and when qualitative feedback from participants was obtained (e.g., through emails, feedback forms, interviews, focus groups).

	1	2	3	4	5	
subitem not at all important	○	○	○	○	○	essential

Does your paper address subitem 6a-iii?

Copy and paste relevant sections from manuscript text

Your answer

6b) Any changes to trial outcomes after the trial commenced, with reasons

Does your paper address CONSORT subitem 6b? *

Copy and paste relevant sections from the manuscript (include quotes in quotation marks "like this" to indicate direct quotes from your manuscript), or elaborate on this item by providing additional information not in the ms, or briefly explain why the item is not applicable/relevant for your study

"There are no outcome changes after trial commencement."

7a) How sample size was determined

NPT: When applicable, details of whether and how the clustering by care provides or centers was addressed

Your response is too large. Try shortening some answers.

7a-i) Describe whether and how expected attrition was taken into account when calculating the sample size

Describe whether and how expected attrition was taken into account when calculating the sample size.

	1	2	3	4	5	
subitem not at all important	○	○	○	○	○	essential

Does your paper address subitem 7a-i?

Copy and paste relevant sections from manuscript title (include quotes in quotation marks "like this" to indicate direct quotes from your manuscript), or elaborate on this item by providing additional information not in the ms, or briefly explain why the item is not applicable/relevant for your study

Your answer

7b) When applicable, explanation of any interim analyses and stopping guidelines

Does your paper address CONSORT subitem 7b? *

Copy and paste relevant sections from the manuscript (include quotes in quotation marks "like this" to indicate direct quotes from your manuscript), or elaborate on this item by providing additional information not in the ms, or briefly explain why the item is not applicable/relevant for your study

"Even though our intervention has no known risk or harm to participants, participants are requested to report any adverse events and withdraw at any point in case any withdrawal Problems happen."

8a) Method used to generate the random allocation sequence

NPT: When applicable, how care providers were allocated to each trial group

Your response is too large. Try shortening some answers.

Does your paper address CONSORT subitem 8a? *

Copy and paste relevant sections from the manuscript (include quotes in quotation marks "like this" to indicate direct quotes from your manuscript), or elaborate on this item by providing additional information not in the ms, or briefly explain why the item is not applicable/relevant for your study

"Allocation sequences were concealed using a computer-generated random number."

8b) Type of randomisation; details of any restriction (such as blocking and block size)

Does your paper address CONSORT subitem 8b? *

Copy and paste relevant sections from the manuscript (include quotes in quotation marks "like this" to indicate direct quotes from your manuscript), or elaborate on this item by providing additional information not in the ms, or briefly explain why the item is not applicable/relevant for your study

"Stratified block randomization was implemented by a trained student assistant (SA) to Allocate participants into intervention and control groups (20 per group). To ensure baseline balance of prognostic factors, particularly ethnicity, sex, and disease severity, which could confound the intervention effects if unevenly distributed, participants were first stratified by these factors. Within each stratum, block randomization (1:1 ratio, block size of 4) was performed using a computer-based random number generator."

9) Mechanism used to implement the random allocation sequence (such as sequentially numbered containers), describing any steps taken to conceal the sequence until interventions were assigned

Your response is too large. Try shortening some answers.

Does your paper address CONSORT subitem 9? *

Copy and paste relevant sections from the manuscript (include quotes in quotation marks "like this" to indicate direct quotes from your manuscript), or elaborate on this item by providing additional information not in the ms, or briefly explain why the item is not applicable/relevant for your study

"computer-generated random number"

10) Who generated the random allocation sequence, who enrolled participants, and who assigned participants to interventions

Does your paper address CONSORT subitem 10? *

Copy and paste relevant sections from the manuscript (include quotes in quotation marks "like this" to indicate direct quotes from your manuscript), or elaborate on this item by providing additional information not in the ms, or briefly explain why the item is not applicable/relevant for your study

"Research assistant (RA)"

11a) If done, who was blinded after assignment to interventions (for example, participants, care providers, those assessing outcomes) and how

NPT: Whether or not administering co-interventions were blinded to group assignment

Your response is too large. Try shortening some answers.

11a-i) Specify who was blinded, and who wasn’t

Specify who was blinded, and who wasn’t. Usually, in web-based trials it is not possible to blind the participants [1, 3] (this should be clearly acknowledged), but it may be possible to blind outcome assessors, those doing data analysis or those administering co-interventions (if any).

	1	2	3	4	5	
subitem not at all important	○	○	○	○	◉	essential

Clear selection

Does your paper address subitem 11a-i? *

Copy and paste relevant sections from the manuscript (include quotes in quotation marks "like this" to indicate direct quotes from your manuscript), or elaborate on this item by providing additional information not in the ms, or briefly explain why the item is not applicable/relevant for your study

"Due to the nature of the intervention and self-administered questionnaires, blinding of participants was not feasible. However, the outcome assessor was blinded to the participants’ group assignments throughout the study."

11a-ii) Discuss e.g., whether participants knew which intervention was the “intervention of interest” and which one was the “comparator”

Informed consent procedures (4a-ii) can create biases and certain expectations - discuss e.g., whether participants knew which intervention was the “intervention of interest” and which one was the “comparator”.

	1	2	3	4	5	
subitem not at all important	○	○	○	○	○	essential

Your response is too large. Try shortening some answers.

Does your paper address subitem 11a-ii?

Copy and paste relevant sections from the manuscript (include quotes in quotation marks "like this" to indicate direct quotes from your manuscript), or elaborate on this item by providing additional information not in the ms, or briefly explain why the item is not applicable/relevant for your study

Your answer

11b) If relevant, description of the similarity of interventions

(this item is usually not relevant for ehealth trials as it refers to similarity of a placebo or sham intervention to a active medication/intervention)

Does your paper address CONSORT subitem 11b? *

Copy and paste relevant sections from the manuscript (include quotes in quotation marks "like this" to indicate direct quotes from your manuscript), or elaborate on this item by providing additional information not in the ms, or briefly explain why the item is not applicable/relevant for your study

"As it is an internet-based intervention, it is not applicable to this study."

12a) Statistical methods used to compare groups for primary and secondary outcomes

NPT: When applicable, details of whether and how the clustering by care providers or centers was addressed

Your response is too large. Try shortening some answers.

Does your paper address CONSORT subitem 12a? *

Copy and paste relevant sections from the manuscript (include quotes in quotation marks "like this" to indicate direct quotes from your manuscript), or elaborate on this item by providing additional information not in the ms, or briefly explain why the item is not applicable/relevant for your study

"The data were analyzed using the IBM SPSS version 23 (IBM, Armonk, NY, USA). Descriptive statistics were employed to summarize the social-demographic characteristics of participants and the feasibility measures. Mean and standard deviation were used for continuous variables with a normal distribution, while medians and interquartile ranges were used for non-normal distributions. Comparability of participants between groups was examined by the independent samples t-test or Mann–Whitney U test for continuous variables and the chi-square test or Fisher's exact test for categorical variables. Additionally, Paired t-tests (for normally distributed data) and Wilcoxon signed-rank tests (for nonnormally distributed data) were used to examine within-subject mean differences. Missing data were handled using intention-to-treat analysis by considering all participants enrolled and randomized to either of the groups."

12a-i) Imputation techniques to deal with attrition / missing values

Imputation techniques to deal with attrition / missing values: Not all participants will use the intervention/comparator as intended and attrition is typically high in ehealth trials. Specify how participants who did not use the application or dropped out from the trial were treated in the statistical analysis (a complete case analysis is strongly discouraged, and simple imputation techniques such as LOCF may also be problematic [4]).

	1	2	3	4	5	
subitem not at all important	○	○	○	○	◉	essential

Clear selection

Your response is too large. Try shortening some answers.

Does your paper address subitem 12a-i? *

Copy and paste relevant sections from the manuscript (include quotes in quotation marks "like this" to indicate direct quotes from your manuscript), or elaborate on this item by providing additional information not in the ms, or briefly explain why the item is not applicable/relevant for your study

"The Generalized Estimating Equations (GEE) method was applied under the assumption that data were missing completely at random (MCAR). However, to preserve the statistical power and reduce bias under the assumption that data were missing at random, GEE with multiple imputations was employed due to the presence of substantial missing data (>5%). missingness in post-test measures)."

12b) Methods for additional analyses, such as subgroup analyses and adjusted analyses

Does your paper address CONSORT subitem 12b? *

Copy and paste relevant sections from the manuscript (include quotes in quotation marks "like this" to indicate direct quotes from your manuscript), or elaborate on this item by providing additional information not in the ms, or briefly explain why the item is not applicable/relevant for your study

"Since the sample size was too small, that made subgroup analysis difficult to perform."

X26) REB/IRB Approval and Ethical Considerations [recommended as subheading under "Methods"] (not a CONSORT item)

X26-i) Comment on ethics committee approval

1 2 3 4 5

Your response is too large. Try shortening some answers.

Does your paper address subitem X26-i?

Copy and paste relevant sections from the manuscript (include quotes in quotation marks "like this" to indicate direct quotes from your manuscript), or elaborate on this item by providing additional information not in the ms, or briefly explain why the item is not applicable/relevant for your study

Your answer

x26-ii) Outline informed consent procedures

Outline informed consent procedures e.g., if consent was obtained offline or online (how? Checkbox, etc.?), and what information was provided (see 4a-ii). See [6] for some items to be included in informed consent documents.

	1	2	3	4	5	
subitem not at all important	○	○	○	○	○	essential

Does your paper address subitem X26-ii?

Copy and paste relevant sections from the manuscript (include quotes in quotation marks "like this" to indicate direct quotes from your manuscript), or elaborate on this item by providing additional information not in the ms, or briefly explain why the item is not applicable/relevant for your study

Your answer

X26-iii) Safety and security procedures

Safety and security procedures, incl. privacy considerations, and any steps taken to reduce the likelihood or detection of harm (e.g., education and training, availability of a hotline)

	1	2	3	4	5	
subitem not at all important	○	○	○	○	○	essential

Your response is too large. Try shortening some answers.

Does your paper address subitem X26-iii?

Copy and paste relevant sections from the manuscript (include quotes in quotation marks "like this" to indicate direct quotes from your manuscript), or elaborate on this item by providing additional information not in the ms, or briefly explain why the item is not applicable/relevant for your study

Your answer

RESULTS

13a) For each group, the numbers of participants who were randomly assigned, received intended treatment, and were analysed for the primary outcome

NPT: The number of care providers or centers performing the intervention in each group and the number of patients treated by each care provider in each center

Does your paper address CONSORT subitem 13a? *

Copy and paste relevant sections from the manuscript (include quotes in quotation marks "like this" to indicate direct quotes from your manuscript), or elaborate on this item by providing additional information not in the ms, or briefly explain why the item is not applicable/relevant for your study

"A total of 148 ethnic minority young adults who drank alcohol were approached from the community. After screening, 38.5% (57/148) met the eligibility criteria. Of them, 85.96% (49/57) consented to join the study. Among these, 81.6% (40/49) were randomly assigned either to the ACT-BA (n = 20) or TAU (n = 20) group. The retention rate was 82.5% (33/40). with 85% (17/20) in the ACT-BA group and 80% (16/20) in the TAU group. The attrition rate was 15% (3/20) for ACT-BA and 20% (4/20) for TAU, but there was no significant difference in attrition between groups (p = .465). Of those who remained in the study, 30 completed the post-intervention test, providing a completion rate of 75% (30/40), with 80% (16/20) in the ACT-BA group and 70% (14/20) in the TAU group."

Your response is too large. Try shortening some answers.

13b) For each group, losses and exclusions after randomisation, together with reasons

Does your paper address CONSORT subitem 13b? (NOTE: Preferably, this is shown in a CONSORT flow diagram) *

Copy and paste relevant sections from the manuscript (include quotes in quotation marks "like this" to indicate direct quotes from your manuscript), or elaborate on this item by providing additional information not in the ms, or briefly explain why the item is not applicable/relevant for your study

"Intervention group: Post-intervention test (n = 16)
• Not available online (n = 2)
• Did not give a response (n=2
The control group: T1: Post-intervention test (n = 14)
• Not available online (n = 2)
• Did not give response (n=4)"

13b-i) Attrition diagram

Strongly recommended: An attrition diagram (e.g., proportion of participants still logging in or using the intervention/comparator in each group plotted over time, similar to a survival curve) or other figures or tables demonstrating usage/dose/engagement.

	1	2	3	4	5	
subitem not at all important	○	○	○	○	○	essential

Does your paper address subitem 13b-i?

Copy and paste relevant sections from the manuscript or cite the figure number if applicable (include quotes in quotation marks "like this" to indicate direct quotes from your manuscript), or elaborate on this item by providing additional information not in the ms, or briefly explain why the item is not applicable/relevant for your study

Your answer

Your response is too large. Try shortening some answers.

14a) Dates defining the periods of recruitment and follow-up

Does your paper address CONSORT subitem 14a? *

Copy and paste relevant sections from the manuscript (include quotes in quotation marks "like this" to indicate direct quotes from your manuscript), or elaborate on this item by providing additional information not in the ms, or briefly explain why the item is not applicable/relevant for your study

"A single-blinded randomized controlled trial study was conducted among Hong Kong ethnic minority young adults with AUD from September 21, 2024, to May 15, 2025, with a total of six weekly individual sessions structured sequentially."

14a-i) Indicate if critical “secular events” fell into the study period

Indicate if critical “secular events” fell into the study period, e.g., significant changes in Internet resources available or “changes in computer hardware or Internet delivery resources”

	1	2	3	4	5	
subitem not at all important	○	○	○	○	○	essential

Does your paper address subitem 14a-i?

Copy and paste relevant sections from the manuscript (include quotes in quotation marks "like this" to indicate direct quotes from your manuscript), or elaborate on this item by providing additional information not in the ms, or briefly explain why the item is not applicable/relevant for your study

Your answer

14b) Why the trial ended or was stopped (early)

Your response is too large. Try shortening some answers.

Does your paper address CONSORT subitem 14b? *

Copy and paste relevant sections from the manuscript (include quotes in quotation marks "like this" to indicate direct quotes from your manuscript), or elaborate on this item by providing additional information not in the ms, or briefly explain why the item is not applicable/relevant for your study

"It is a pilot trial designed to examine preliminary effectiveness and feasibility postintervention. Therefore, intervention was completed at 6 weeks of intervention."

15) A table showing baseline demographic and clinical characteristics for each group

NPT: When applicable, a description of care providers (case volume, qualification, expertise, etc.) and centers (volume) in each group

Does your paper address CONSORT subitem 15? *

Copy and paste relevant sections from the manuscript (include quotes in quotation marks "like this" to indicate direct quotes from your manuscript), or elaborate on this item by providing additional information not in the ms, or briefly explain why the item is not applicable/relevant for your study

"The study participants' socio-demographic characteristics are presented in Table 1. Participants had a mean age of 32.43 years (SD = 2.57), with 80% female and 87.7% Filipino. Eighty-five percent were Catholic, 52.5% were single, and 85% were employed. Of those being employed, 87.5% were domestic helpers. Approximately 60% attained primary or secondary education, and 32.5% cohabited with partners. The median duration of residence in Hong Kong was 5 years, with an Interquartile Range (IQR) of 2-6. Regarding the AUD severity, 47.5% had moderate AUD, 32.5% had severe AUD, and 20% had mild AUD. Baseline comparisons between groups revealed no significant differences in demographics and the AUD severity."

Your response is too large. Try shortening some answers.

15-i) Report demographics associated with digital divide issues

In ehealth trials it is particularly important to report demographics associated with digital divide issues, such as age, education, gender, social-economic status, computer/Internet/ehealth literacy of the participants, if known.

	1	2	3	4	5	
subitem not at all important	○	○	○	○	○	essential

Does your paper address subitem 15-i? *

Copy and paste relevant sections from the manuscript (include quotes in quotation marks "like this" to indicate direct quotes from your manuscript), or elaborate on this item by providing additional information not in the ms, or briefly explain why the item is not applicable/relevant for your study

We recruited participants with experience in using smartphones and the internet."

16) For each group, number of participants (denominator) included in each analysis and whether the analysis was by original assigned groups

16-i) Report multiple “denominators” and provide definitions

Report multiple “denominators” and provide definitions: Report N’s (and effect sizes) “across a range of study participation [and use] thresholds” [1], e.g., N exposed, N consented, N used more than x times, N used more than y weeks, N participants “used” the intervention/comparator at specific pre-defined time points of interest (in absolute and relative numbers per group). Always clearly define “use” of the intervention.

	1	2	3	4	5	
subitem not at all important	○	○	○	○	◉	essential

Clear selection

Your response is too large. Try shortening some answers.

Does your paper address subitem 16-i? *

Copy and paste relevant sections from the manuscript (include quotes in quotation marks "like this" to indicate direct quotes from your manuscript), or elaborate on this item by providing additional information not in the ms, or briefly explain why the item is not applicable/relevant for your study

"A total of 148 ethnic minority young adults who drank alcohol were approached from the community. After screening, 38.5% (57/148) met the eligibility criteria. Of them, 85.96% (49/57) consented to join the study. Among these, 81.6% (40/49) were randomly assigned either to the ACT-BA (n = 20) or TAU (n = 20) group. The retention rate was 82.5% (33/40). with 85% (17/20) in the ACT-BA group and 80% (16/20) in the TAU group. The attrition rate was 15% (3/20) for ACT-BA and 20% (4/20) for TAU, but there was no significant difference in attrition between groups (p = .465). Of those who remained in the study, 30 completed the post-intervention test, providing a completion rate of 75% (30/40), with 80% (16/20) in the ACT-BA group and 70% (14/20) in the TAU group."

16-ii) Primary analysis should be intent-to-treat

Primary analysis should be intent-to-treat, secondary analyses could include comparing only “users”, with the appropriate caveats that this is no longer a randomized sample (see 18-i).

	1	2	3	4	5	
subitem not at all important	○	○	○	○	●	essential

Clear selection

Your response is too large. Try shortening some answers.

Does your paper address subitem 16-ii?

Copy and paste relevant sections from the manuscript (include quotes in quotation marks "like this" to indicate direct quotes from your manuscript), or elaborate on this item by providing additional information not in the ms, or briefly explain why the item is not applicable/relevant for your study

"Missing data were handled using intention-to-treat analysis by considering all participants enrolled and randomized to either of the groups. Little's test was used to determine whether the missing data was completely at random (MCAR) or not. The Generalized Estimating The equations (GEE) method was applied under the assumption that data were missing completely at random (MCAR)."

17a) For each primary and secondary outcome, results for each group, and the estimated effect size and its precision (such as 95% confidence interval)

Does your paper address CONSORT subitem 17a? *

Copy and paste relevant sections from the manuscript (include quotes in quotation marks "like this" to indicate direct quotes from your manuscript), or elaborate on this item by providing additional information not in the ms, or briefly explain why the item is not applicable/relevant for your study

"The ACT-BA intervention provided a moderate effect size on drinking days (Cohen's d = -0.57) and a large effect size on drinks per drinking day (Cohen's d = -1.89), in cumulative abstinence duration (Cohen's d = 0.32), and in heavy drinking days (Cohen's d = -0.71), alcohol abstinence self-efficacy (Cohen's d = 0.81), psychological flexibility (Cohen's d = -0.65), and readiness to change (Cohen's d = 0.28) and everyday discrimination. (Cohen's d = -0.48)."

Your response is too large. Try shortening some answers.

17a-i) Presentation of process outcomes such as metrics of use and intensity of use

In addition to primary/secondary (clinical) outcomes, the presentation of process outcomes such as metrics of use and intensity of use (dose, exposure) and their operational definitions is critical. This does not only refer to metrics of attrition (13-b) (often a binary variable), but also to more continuous exposure metrics such as “average session length”. These must be accompanied by a technical description how a metric like a “session” is defined (e.g., timeout after idle time) [1] (report under item 6a).

	1	2	3	4	5	
subitem not at all important	○	○	○	○	○	essential

Does your paper address subitem 17a-i?

Copy and paste relevant sections from the manuscript (include quotes in quotation marks "like this" to indicate direct quotes from your manuscript), or elaborate on this item by providing additional information not in the ms, or briefly explain why the item is not applicable/relevant for your study

Your answer

17b) For binary outcomes, presentation of both absolute and relative effect sizes is recommended

Does your paper address CONSORT subitem 17b? *

Copy and paste relevant sections from the manuscript (include quotes in quotation marks "like this" to indicate direct quotes from your manuscript), or elaborate on this item by providing additional information not in the ms, or briefly explain why the item is not applicable/relevant for your study

"Our outcome variable was a continuous variable. Therefore, it is not applicable."

Your response is too large. Try shortening some answers.

18) Results of any other analyses performed, including subgroup analyses and adjusted analyses, distinguishing pre-specified from exploratory

Your response is too large. Try shortening some answers.

Does your paper address CONSORT subitem 18? *

Copy and paste relevant sections from the manuscript (include quotes in quotation marks "like this" to indicate direct quotes from your manuscript), or elaborate on this item by providing additional information not in the ms, or briefly explain why the item is not applicable/relevant for your study

Your response is too large. Try shortening some answers.

"The ACT-BA intervention showed a significant group-by-time interaction effect on various drinking outcomes. Compared to the TAU group, participants in the intervention group showed a statistically significant reduction in drinking days (ITT: Group*time, B = -4.12, 95% Confidence Interval [CI]: -8.10, -0.13, Wald χ^2 (4) = 4.224, P = .043) and (PP: Group*time, B = -4.02, 95% CI: -7.50, -0.54, Wald χ^2 (1) = 5.122.

, P = .024) and drinks per drinking day (ITT: Group*time, B = -1.56, 95% CI: -3.06, -0.07, Wald χ^2 (4) = 4.298, P P= .041) and (PP: Group*time, B = -1.31, 95% CI: -2.60, -0.02, Wald χ^2 (1) = 3.956, P = .047) at post-intervention. The ACT-BA intervention provided a moderate effect size on drinking days (Cohen's d = -0.57) and a large effect size on drinks per drinking day (Cohen's d = -1.89) (Table 4). Although a statistical significant difference was not reached, However, there were no significant between-group differences in cumulative abstinence duration (ITT; Group*time, B = 2.34, 95% CI: -3.68, 8.36, Wald χ^2 (4) = .634, P = .433) and (PP; Group*time, B = 2.36, 95% CI: -2.45, 7.17, Wald χ^2 (1) = .925, P = .336) and heavy drinking days (ITT: Group*time, B = -3.01, 95% CI: -6.37, -0.36, Wald χ^2 (4) = 3.135, P = .079) and (PP: Group*time, B = -3.58, 95% CI: -7.19, 0.02, Wald χ^2 (1) = 3.794, P = .051) at post-intervention assessment (Table 5). The ACT-BA group showed improvement with small-to-moderate (Cohen's d = 0.32) and moderate-to-large (Cohen's d = -0.71) effect sizes, respectively.

Effectiveness of ACT-BA on AASE

A significant improvement in alcohol abstinence self-efficacy was observed in the intervention group compared to those in the control group at post-intervention. Participants who received the ACT-BA intervention showed a statistically significant increase in the mean AAES score from baseline to post-intervention in both ITT and PP analysis (ITT: Group*time, B = 11.95, 95% CI: 0.10, 23.81, Wald χ^2 (4) = 3.960, P = .048) and PP analysis (Group*time, B = 12.71, 95% CI: 2.79, 22.64, Wald χ^2 (1) = 6.306, P = .012) (Table 5). The effect size of the intervention on alcohol abstinence self-efficacy was also large in both analyses, ITT (Cohen's d = 0.81) and PP (Cohen's d = 0.83) (Table 4).

Effectiveness of ACT-BA on psychological flexibility

Participants in the intervention group revealed a statistically significant reduction in the mean score of AAQ-II at post-intervention in both analyses (ITT: Group*time, B = -6.41, 95% CI: -12.77, -0.06, Wald χ^2 (4) = 3.960, P = .041) (PP: Group*time, B = -6.93, 95% CI: (-11.95, -1.92, Wald χ^2 (1) = 7.333, P = .001). The effect size on psychological flexibility was moderate (ITT: Cohen's d = -0.65) and (PP: Cohen's d = -0.7) (Table 4x).

Effectiveness of ACT-BA on readiness to change

A mild improvement was observed in the mean score of RTCQ score in the intervention group, with a mild effect size (Cohen's d = 0.28) (Table 4). However, this change did not reach a statistically significant difference in this study in both analyses (ITT: Group*time, B = 2.71, 95% CI: -6.80, 12.23, Wald χ^2 (4) = .317, P = .574) and (PP: Group*time, B = 2.50, 95% CI: -7.79, 12.79, Wald χ^2 (1) = .227, P = .227) (Table 5), with a small effect size in both analyses (Cohen's d = 0.28) (Table 4).

Effectiveness of ACT-BA on everyday discrimination

As presented in Table 5, a greater improvement in the mean score of everyday discrimination was observed in the ACT-BA group compared to the TAU group at post-intervention. Nevertheless, the GEE (Table 5) revealed no statistically significant difference between the two groups (ITT: Group*time, B = -4.04, 95% CI: -10.43, 2.35, Wald χ^2 (4) = 1.533, P = .216) and (PP: Group*time, B = -5.26, 95% CI: -11.84, 1.32, Wald χ^2 (1) = 2.458, P = .117),

Your response is too large. Try shortening some answers.

18-i) Subgroup analysis of comparing only users

A subgroup analysis of comparing only users is not uncommon in ehealth trials, but if done, it must be stressed that this is a self-selected sample and no longer an unbiased sample from a randomized trial (see 16-iii).

	1	2	3	4	5	
subitem not at all important	○	○	○	○	○	essential

Does your paper address subitem 18-i?

Copy and paste relevant sections from the manuscript (include quotes in quotation marks "like this" to indicate direct quotes from your manuscript), or elaborate on this item by providing additional information not in the ms, or briefly explain why the item is not applicable/relevant for your study

Your answer

19) All important harms or unintended effects in each group

(for specific guidance see CONSORT for harms)

Does your paper address CONSORT subitem 19? *

Copy and paste relevant sections from the manuscript (include quotes in quotation marks "like this" to indicate direct quotes from your manuscript), or elaborate on this item by providing additional information not in the ms, or briefly explain why the item is not applicable/relevant for your study

"They were informed of their right to decline or withdraw from the study at any time if any harm or discomfort."

Your response is too large. Try shortening some answers.

19-i) Include privacy breaches, technical problems

Include privacy breaches, technical problems. This does not only include physical “harm” to participants, but also incidents such as perceived or real privacy breaches [1], technical problems, and other unexpected/unintended incidents. “Unintended effects” also includes unintended positive effects [2].

	1	2	3	4	5	
subitem not at all important	○	○	○	○	○	essential

Does your paper address subitem 19-i?

Copy and paste relevant sections from the manuscript (include quotes in quotation marks "like this" to indicate direct quotes from your manuscript), or elaborate on this item by providing additional information not in the ms, or briefly explain why the item is not applicable/relevant for your study

Your answer

19-ii) Include qualitative feedback from participants or observations from staff/researchers

Include qualitative feedback from participants or observations from staff/researchers, if available, on strengths and shortcomings of the application, especially if they point to unintended/unexpected effects or uses. This includes (if available) reasons for why people did or did not use the application as intended by the developers.

	1	2	3	4	5	
subitem not at all important	○	○	○	○	○	essential

Your response is too large. Try shortening some answers.

Does your paper address subitem 19-ii?

Copy and paste relevant sections from the manuscript (include quotes in quotation marks "like this" to indicate direct quotes from your manuscript), or elaborate on this item by providing additional information not in the ms, or briefly explain why the item is not applicable/relevant for your study

Your answer

DISCUSSION

22) Interpretation consistent with results, balancing benefits and harms, and considering other relevant evidence

NPT: In addition, take into account the choice of the comparator, lack of or partial blinding, and unequal expertise of care providers or centers in each group

22-i) Restate study questions and summarize the answers suggested by the data, starting with primary outcomes and process outcomes (use)

Restate study questions and summarize the answers suggested by the data, starting with primary outcomes and process outcomes (use).

	1	2	3	4	5	
subitem not at all important	○	○	○	○	◉	essential

Clear selection

Your response is too large. Try shortening some answers.

Does your paper address subitem 22-i? *

Copy and paste relevant sections from the manuscript (include quotes in quotation marks "like this" to indicate direct quotes from your manuscript), or elaborate on this item by providing additional information not in the ms, or briefly explain why the item is not applicable/relevant for your study

Your response is too large. Try shortening some answers.

"This study was conducted among Hong Kong ethnic minority young adults with AUD, a population that often encounters barriers to healthcare access and has low treatment engagement and intervention adherence due to low risk perception or poor health literacy (97, 98). To address this problem, we designed and evaluated a novel internet-based self-help program integrating ACT and BA among Hong Kong ethnic minority young adults with AUD.

The present study provides preliminary evidence that our proposed intervention statistically significantly improved psychological flexibility, with a moderate effect size among ethnic minority young adults with AUD. This finding is consistent with previous studies on ACT for AUD (90, 99). However, our effect size on psychological flexibility is larger than those in previous studies, ranging from small to moderate (100). The larger effect size might be due to the integration of ACT and BA. Particularly, ACT emphasizes enhancing psychological flexibility in which participants learn different skills to allow difficult thoughts, feelings, and sensations, thus reducing experiential avoidance, which might trigger drinking as a coping behavior (50). BA, as an additional component, further boosted the participants' psychological flexibility by guiding them to identify a list of alternatives and value congruent activities to replace drinking (101).

Our previous cross-sectional study and qualitative interviews identified that experiences of discrimination were a key factor leading to the development and maintenance of AUD among ethnic minority young adults (29). Our quantitative findings showed a moderate effect on discrimination, revealing its potential efficacy in reducing the mean score of discrimination experiences, although a statistically significant mean difference was not reached between groups at post-intervention. Nevertheless, our quantitative findings suggested that the ACT-BA intervention might not have a significant effect on this variable, as there was no statistically significant difference in the mean everyday discrimination score between the intervention and control groups at post-intervention. The insignificant finding of difference between groups could be that our intervention did not aim to reduce the discrimination encountered by the participants. Instead, the core focus of the intervention was to enhance the participants' psychological flexibility to cope with the discrimination (50). As such, the intervention might not have a significant effect on the participants' everyday discrimination, which was captured by the EDS as an outcome. Another possible explanation for the insignificant finding difference is that the sample size of this study was small, resulting in a type II error in the analysis (102).

The ACT-BA intervention successfully enhanced participants' alcohol abstinence self-efficacy in the intervention group compared to the TAU group, with a large effect size at post-intervention. Alcohol abstinence self-efficacy is defined as personal confidence in their own ability to abstain from alcohol (103). Bandura (1977) pointed out that self-efficacy is an important construct in determining whether a person will have attempts and take actions for behavioral changes (104). Although our intervention did not contain any strategy that could directly improve self-efficacy in alcohol abstinence among our participants, it can be indirectly and gradually built up in our intervention through participants' successful experience in alcohol reduction when they try to cut down the use of drinking (105). Based on this preliminary finding, we expect that the intervention effect on alcohol abstinence self-efficacy would be stronger at a later time because of more successful experience in alcohol reduction. This postulation shall be further confirmed by a full-scale RCT with longer follow-[illegible]

Your response is too large. Try shortening some answers.

[illegible] on some drinking outcomes. Particularly, compared to the TAU group, participants in the [illegible]

intervention group reported an average reduction of 4.12 drinking days per month and 1.15 drinks per drinking day at post-intervention, with -0.57 and -1.89 effect sizes, respectively. These reductions are perhaps a reflection of the synergetic efficacy of the integrated intervention on the participants' psychological flexibility, which emphasized noticing negative and unfavorable feelings without judgment while adapting alternative behaviors to replace drinking in view of pursuing the life goals (106). Notwithstanding the positive intervention effects and statistically significant mean difference on the number of drinking days and number of drinks per drinking day, we did not find any statistically significant effect difference on the number of heavy drinking days and cumulative alcohol abstinence. Despite no statistically significant difference between groups, the intervention group The insignificant effect mean difference for these two outcomes was likely because complete abstinence from alcohol could trigger withdrawal symptoms, which were not heavily emphasized in our intervention, and these withdrawal symptoms were hard to be overcome by only improving their psychological flexibility and self-efficacy in alcohol abstinence. This explanation is also supported by our qualitative findings that some participants in the experimental group reported difficulties in managing the withdrawal symptoms, e.g., agitation and increased heart and respiratory rates, even though they were motivated to quit alcohol use after the intervention. In response to this issue, this intervention can, on one hand, be strengthened by increasing the content about the management of withdrawal symptoms and, on the other hand, by referring the participants who may require medications for withdrawal symptoms to healthcare professionals alongside the intervention. Another possible explanation for the insignificant findings was that reduction in heavy drinking and alcohol abstinence are more distal outcomes, which could only be captured and detected by a longer follow-up, which is six months, a widely accepted international standard for evaluation of behavioral changes (107). Thus, a follow-up duration of 6 months or more is recommended for future full-scale RCTs.

"

22-ii) Highlight unanswered new questions, suggest future research

Highlight unanswered new questions, suggest future research.

	1	2	3	4	5	
subitem not at all important	○	○	○	○	◉	essential

Clear selection

Your response is too large. Try shortening some answers.

Does your paper address subitem 22-ii?

Copy and paste relevant sections from the manuscript (include quotes in quotation marks "like this" to indicate direct quotes from your manuscript), or elaborate on this item by providing additional information not in the ms, or briefly explain why the item is not applicable/relevant for your study

"This study addresses the literature gaps by providing evidence on the preliminary effectiveness and feasibility of the ACT-BA on different drinking-related outcomes among ethnic minorities with AUD. Therefore, a key implication for future study was to conduct a fully-powered RCT to further examine the effectiveness of such intervention, with longer follow-up to capture the long-term effect on different drinking outcomes and incorporating objective measures to assess the drinking outcomes to minimize the social desirability bias. Given that the intervention can be self-administered in an online platform with only a minimal involvement from interventionalists, a flexible schedule for the working group, and at low cost, it is expected that this intervention, if proven to be effective in a large-scale RCT, can be delivered to a large group of ethnic minority young adults in the community."

20) Trial limitations, addressing sources of potential bias, imprecision, and, if relevant, multiplicity of analyses

20-i) Typical limitations in ehealth trials

Typical limitations in ehealth trials: Participants in ehealth trials are rarely blinded. Ehealth trials often look at a multiplicity of outcomes, increasing risk for a Type I error. Discuss biases due to non-use of the intervention/usability issues, biases through informed consent procedures, unexpected events.

	1	2	3	4	5	
subitem not at all important	○	○	○	○	◉	essential

Clear selection

Your response is too large. Try shortening some answers.

Does your paper address subitem 20-i? *

Copy and paste relevant sections from the manuscript (include quotes in quotation marks "like this" to indicate direct quotes from your manuscript), or elaborate on this item by providing additional information not in the ms, or briefly explain why the item is not applicable/relevant for your study

"This RCT has several limitations. Firstly, the small sample size may reduce the statistical power, potentially affecting the true effect size of the interventions. Secondly, the self-help intervention limited direct measurement of the participants' engagement with exercises, metaphors, and practices of each module. Nevertheless, engagement was assessed indirectly through online tracking of the module completion time and daily exercise logs. Thirdly, the preliminary effectiveness was assessed immediately post-intervention. Although psychosocial interventions usually bring behavioral changes over time following the intervention, the absence of follow-up assessment may limit the evaluation of the long-term effect of the ACT-BA. Fourthly, this study relied solely on participants' self-reported data rather than objective assessments, which might introduce bias and potentially lead to an over- or underestimation of the effect size of the outcome variables. Fifthly, although the ACT-BA showed significant effects across multiple outcomes, how the effects varied across different ethnic minority groups was not examined. Sixth, although almost all ethnic minority participants were able to speak English, excluding individuals who do not speak English may have introduced selection bias, potentially limiting the generalizability of findings to all ethnic minority groups."

21) Generalisability (external validity, applicability) of the trial findings

NPT: External validity of the trial findings according to the intervention, comparators, patients, and care providers or centers involved in the trial

21-i) Generalizability to other populations

Generalizability to other populations: In particular, discuss generalizability to a general Internet population, outside of a RCT setting, and general patient population, including applicability of the study results for other organizations

	1	2	3	4	5	
subitem not at all important	○	○	○	○	○	essential

Your response is too large. Try shortening some answers.

Does your paper address subitem 21-i?

Copy and paste relevant sections from the manuscript (include quotes in quotation marks "like this" to indicate direct quotes from your manuscript), or elaborate on this item by providing additional information not in the ms, or briefly explain why the item is not applicable/relevant for your study

Your answer

21-ii) Discuss if there were elements in the RCT that would be different in a routine application setting

Discuss if there were elements in the RCT that would be different in a routine application setting (e.g., prompts/reminders, more human involvement, training sessions or other co-interventions) and what impact the omission of these elements could have on use, adoption, or outcomes if the intervention is applied outside of a RCT setting.

	1	2	3	4	5	
subitem not at all important	○	○	○	○	○	essential

Does your paper address subitem 21-ii?

Copy and paste relevant sections from the manuscript (include quotes in quotation marks "like this" to indicate direct quotes from your manuscript), or elaborate on this item by providing additional information not in the ms, or briefly explain why the item is not applicable/relevant for your study

Your answer

OTHER INFORMATION

23) Registration number and name of trial registry

Your response is too large. Try shortening some answers.

Does your paper address CONSORT subitem 23? *

Copy and paste relevant sections from the manuscript (include quotes in quotation marks "like this" to indicate direct quotes from your manuscript), or elaborate on this item by providing additional information not in the ms, or briefly explain why the item is not applicable/relevant for your study

"ClinicalTrials.gov NCT06779006 at ClinicalTrials.gov"

24) Where the full trial protocol can be accessed, if available

Does your paper address CONSORT subitem 24? *

Cite a Multimedia Appendix, other reference, or copy and paste relevant sections from the manuscript (include quotes in quotation marks "like this" to indicate direct quotes from your manuscript), or elaborate on this item by providing additional information not in the ms, or briefly explain why the item is not applicable/relevant for your study

"ClinicalTrials.gov NCT06779006; https://clinicaltrials.gov/study/NCT06779006"

25) Sources of funding and other support (such as supply of drugs), role of funders

Does your paper address CONSORT subitem 25? *

Copy and paste relevant sections from the manuscript (include quotes in quotation marks "like this" to indicate direct quotes from your manuscript), or elaborate on this item by providing additional information not in the ms, or briefly explain why the item is not applicable/relevant for your study

"No external financial support or grants were received from any public, commercial, or not-for-profit entities for the research, authorship, or publication of this article."

Your response is too large. Try shortening some answers.

X27-i) State the relation of the study team towards the system being evaluated

In addition to the usual declaration of interests (financial or otherwise), also state the relation of the study team towards the system being evaluated, i.e., state if the authors/evaluators are distinct from or identical with the developers/sponsors of the intervention.

	1	2	3	4	5	
subitem not at all important	○	○	○	○	◉	essential

Clear selection

Does your paper address subitem X27-i?

Copy and paste relevant sections from the manuscript (include quotes in quotation marks "like this" to indicate direct quotes from your manuscript), or elaborate on this item by providing additional information not in the ms, or briefly explain why the item is not applicable/relevant for your study

Your answer

About the CONSORT EHEALTH checklist

As a result of using this checklist, did you make changes in your manuscript? *

- ○ yes, major changes
- ○ yes, minor changes
- ◉ no

Your response is too large. Try shortening some answers.

What were the most important changes you made as a result of using this checklist?

Your answer

How much time did you spend on going through the checklist INCLUDING making changes in your manuscript *

"I have spent 5 hours completing the form."

As a result of using this checklist, do you think your manuscript has improved? *

- (x) yes
- ( ) no
- ( ) Other:

Would you like to become involved in the CONSORT EHEALTH group?

This would involve for example becoming involved in participating in a workshop and writing an "Explanation and Elaboration" document

- ( ) yes
- (x) no
- ( ) Other:

Clear selection

Your response is too large. Try shortening some answers.

Any other comments or questions on CONSORT EHEALTH

Your answer

STOP - Save this form as PDF before you click submit

To generate a record that you filled in this form, we recommend to generate a PDF of this page (on a Mac, simply select "print" and then select "print as PDF") before you submit it.

When you submit your (revised) paper to JMIR, please upload the PDF as supplementary file.

Don't worry if some text in the textboxes is cut off, as we still have the complete information in our database. Thank you!

Final step: Click submit !

Click submit so we have your answers in our database!

Submit Clear form

Never submit passwords through Google Forms.

This content is neither created nor endorsed by Google. - Contact form owner - Terms of Service - Privacy Policy

Does this form look suspicious? Report

Google Forms

Your response is too large. Try shortening some answers.

Your response is too large. Try shortening some answers.
